# Utilising activity patterns of a complex biophysical network model to optimise intra-striatal deep brain stimulation

**DOI:** 10.1038/s41598-024-69456-7

**Published:** 2024-08-14

**Authors:** Konstantinos Spiliotis, Revathi Appali, Anna Karina Fontes Gomes, Jan Philipp Payonk, Simon Adrian, Ursula van Rienen, Jens Starke, Rüdiger Köhling

**Affiliations:** 1 Institute of Mathematics, University of Rostock, Rostock, Germany; 2 Institute of General Electrical Engineering, University of Rostock, Rostock, Germany; 3 Department of Life, Light and Matter, University of Rostock, Rostock, Germany; 4 Department of Ageing of Individuals and Society, University of Rostock, Rostock, Germany; 5 Oscar-Langendorff-Institute of Physiology, Rostock University Medical Center, Rostock, Germany; 6 Faculty of Computer Science and Electrical Engineering, University of Rostock, Rostock, Germany; 7 Laboratory of Mathematics and Informatics (ISCE), Department of Civil Engineering, Democritus University of Thrace, Xanthi, Greece

**Keywords:** Neuronal network dynamics, Striatum, Deep brain stimulation (DBS), Mental disorders, Mathematics and computing, Applied mathematics, Computational models, Computational neuroscience, Computational neuroscience, Biomedical engineering, Control theory, Dynamical systems, Nonlinear dynamics, Systems analysis

## Abstract

A large-scale biophysical network model for the isolated striatal body is developed to optimise potential intrastriatal deep brain stimulation applied to, e.g. obsessive-compulsive disorder. The model is based on modified Hodgkin–Huxley equations with small-world connectivity, while the spatial information about the positions of the neurons is taken from a detailed human atlas. The model produces neuronal spatiotemporal activity patterns segregating healthy from pathological conditions. Three biomarkers were used for the optimisation of stimulation protocols regarding stimulation frequency, amplitude and localisation: the mean activity of the entire network, the frequency spectrum of the entire network (rhythmicity) and a combination of the above two. By minimising the deviation of the aforementioned biomarkers from the normal state, we compute the optimal deep brain stimulation parameters, regarding position, amplitude and frequency. Our results suggest that in the DBS optimisation process, there is a clear trade-off between frequency synchronisation and overall network activity, which has also been observed during in vivo studies.

## Introduction

The striatum constitutes a subcortical region which loops information from the cortex via the other basal ganglia nuclei and the thalamus back to the cortex, thereby orchestrating such varied activities as motor control, decision-making, choosing actions, and, importantly, also reward behaviour^1–3^. The striatum integrates cortical signals (prefrontal, motor, cerebral cortex) to create motor activities based on experience and forthcoming selections. Structurally, the caudate nucleus and the putamen form the striatal structure. On a cellular level, the striatum mainly consists of GABAergic medium spiny neurons (MSN) (95%g), primarily projecting out of the striatum, with collaterals to other MSNs, and interneurons (5%, most of these parvalbumin-positive)^4–6^.

The striatum has long been known to be involved in several neurological diseases resulting in movement disorders, ranging from Parkinson’s disease (PD) via Dystonias to Huntington’s disease (HD) as a widespread neurodegenerative disorder. Notably, the striatum also impacts cognitive and reward processes (particularly the dorsal striatum) and hence, striatal function has been recognised to be pivotal in psychiatric conditions such as obsessive-compulsive disorder (OCD), depression, impulsivity, and attention-deficit hyperactivity disorder (ADHD)^3,7^. Deep brain stimulation of the striatum has therefore been introduced relatively recently as a novel approach for the treatment of OCD^8–10^.

It is long known that dopaminergic projections from the substantia nigra are essential for striatal functionality, affecting both the direct and indirect pathways^11,12^ (activating the former via D1 and dampening the latter via D2 receptors), and thus are crucially involved in the initiation of movements. A substantial part of the dopaminergic projections to the striatum (in particular the dorsal striatum), however, also comes from the ventral tegmental area (VTA)^13,14^, which also projects to the nucleus accumbens, amygdala and prefrontal cortex. The dorsal striatum, in turn, projects to similar areas: the amygdala and the prefrontal cortex via the dorsal thalamic nuclei. In this way, the dorsal striatum has been recognised to be particularly involved in decision-making, goal-directed actions and reward mechanisms^15^. Consequently, low dopamine levels and disturbed striatal activity are linked with diseases involving movement disorders, but also depression and other neuropsychiatric diseases^2,16,17^.

**Figure 1:**
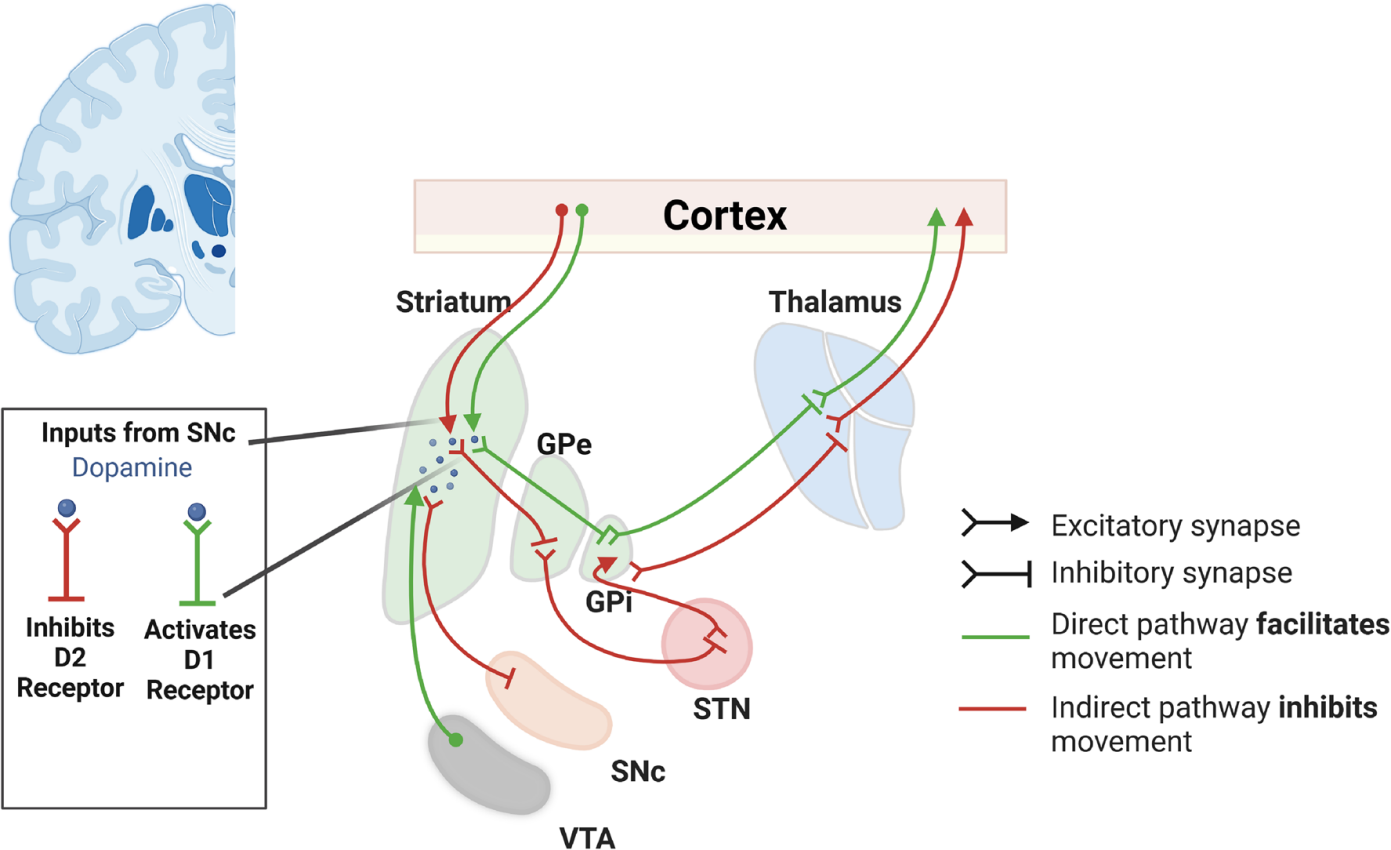
Pathways and circuits of basal ganglia. The basal ganglia circuit contains deep brain areas: the subthalamic nucleus (STN), the globus pallidus external and internal (GPe/GPi), the substantia nigra pars compacta (SNC) and the striatum. The striatum constitutes an intermediate hub between cortex and basal ganglia. Specifically, from the striatum passes the direct pathways (which facilitate the movement depicted in green lines) and the indirect pathway (which inhibits or controls the end of the movement and is represented with red streaks). The arrowheads indicate the excitatory connections, and the line heads illustrate the inhibitory connections. Another deep region that projects to the striatum is the ventral tegmental area (VTA).

The main other input to the striatum is, obviously, cortical. This is provided as glutamatergic, excitatory cortico-striatal projections to medium spiny neurons^18^. Glutamatergic innervation to the striatum mainly projecting from cerebral and frontal cortex^19^ also sets the activity level of GABAergic neurons in the way of feed-forward inhibition and modulates striatal outputs controlling motor behaviour refer Fig. 1. Additionally, glutaminergic activation (through NMDA and AMPA receptors) regulates the formation of the synapses^19,20^.

Pathological conditions in the basal ganglia network are usually accompanied by changes in glutamatergic and dopaminergic signalling, suggesting that the dynamic interaction of these two inputs into the striatum becomes complex in disease. Postmortem studies in tissue from the caudate and putamen of patients with PD (compared to tissue from persons with causes of death unrelated to the brain) showed a reduction of about 27% in dendritic spines of MSNs^21^. Additionally, the MSN neurons in the caudate nucleus displayed a significant length reduction in PD. Dendritic spines receive crucial excitatory input from the cortex; thus, the spine density reduction in conjunction with the total dendritic length decrement was thought to have a negative impact on the excitatory tone of MSN neurons^21^.

Similar conclusions can be drawn more generally from the classical model of basal ganglia activity^11,22,23^. Specifically, the classical model of the basal ganglia predicts that loss of striatal dopamine will decrease extracellular levels of glutamate in the striatum and cortex^11,22^. Abnormalities in these dopaminergic and glutamatergic systems have been observed in numerous disorders, including Parkinson’s disease^24^, and beyond movement disorders, also in depression, OCD and schizophrenia^25,26^. Notably, previous fMRI studies^7,27^ in patients with OCD showed decreased responsiveness and activity in the ventral striatal and caudate. Conversely, healthy controls had increased perfusion in all striatal areas compared to the patients^7^.

Further studies suggest that the glutamatergic and dopaminergic input interact complexly beyond the basal ganglia^23,28–30^. Thus, it has been shown that NMDA receptor activation regulates D1-dopamine receptor signalling in cortical neurons and vice versa^28–30^. Specifically, activation of D1-receptors in the prefrontal cortical pyramidal neurons by the agonist SKF81297 increases the stationary NMDA evoked current^29^. Inversely, activation of NMDA receptors by glutamate results in the recruitment of D1-receptors in cortical neurons, while they do not affect the distribution of D2-receptors^28^. Taking these findings together, we hypothesise that any lesion of the glutaminergic-dopaminergic circuit leads to malfunctioning and, consequently, reduced striatal activity.

Deep brain stimulation (DBS) of the striatum has evolved as a promising therapy for patients with severe and resistant forms of OCD and mental impairments^31–33^. As in the case of DBS for PD, considerable unknowns remain, including the anatomical targets of stimulation, optimal stimulation parameters, long-term effects of stimulation, and the patient’s clinical and biological response to DBS. Progress in predicting therapeutic DBS effects (by optimising DBS parameters: position, intensity, frequency, etc.) was achieved using different computational models^34–39^. However, due to the strongly heterogeneous nature of the connection topology and intrinsic complexity (stochastic and nonlinear large scale neuron, multiple scales)^40–45^, DBS outcome is far from trivial to predict.

Towards this direction, we extend a previous model of the striatal activity^46^, completing the DBS action in the mathematical model. Specifically, the contribution of this work is two-fold. (i) It presents a large-scale biophysical model of the striatum to predict neural activity and spatial-temporal patterns, and, using the model, (ii) it optimises DBS parameters (namely, placement of electrodes, frequency, and amplitude).

We obtain (i) by modelling the neurons with modified Hodgkin–Huxley equations^46,47^ and using a complex graph structure to model the neural network. We construct the graph structure from the real coordinates of a human atlas^48^, placing two types of striatal neurons: interneurons as fast-spiking (FS) and MSN neurons. Going beyond other studies published before^46^, we use complex connectivity (i.e., small world structures^49^) simulating more realistic neuron connectivity structures^44,50^. Integrating this high-dimensional nonlinear system, we produce spatiotemporal patterns of striatal activity. Our model predicts, similar to the classical model of abnormal activity of basal ganglia^22,23^, reduced striatal activity when a cortico-striatal connectivity breakdown is simulated, which renders it a candidate for deriving optimised DBS parameters.

(ii) To obtain optimised DBS parameters, we use three different macroscopic measures or biomarkers: (a) The mean network activity of the striatum, (b) the rhythmicity of the entire striatum network computed as Fourier spectrum of the mean membrane potential of striatum neurons (c) a combination of the mean activity with the rhythmicity of the network.

Based on the deviation of the indices from the healthy state, we propose a DBS protocol that provides the therapeutic pattern for abnormal striatum network activity. Unlike other models, we include a sensitivity analysis gauging the impact of specific parameters on total network activity and corroborating our assumption that individual parameters play a paramount role.

## Materials and methods

### Data sources

The structural Magnetic Resonance Imaging (MRI) data are taken from the Human Connectome Project (HCP)^51^. These MRI data were laid over another atlas, the MNIPD25 atlas, which is most commonly used for surgery planning^52–54^ with the help of Lead-DBS, a toolbox for atlas connectivity and segregation analyses^55^. The structural composition striatum, in turn, is taken from the MIDA atlas^48^ and translated into the MNI (Montreal Neurological Institute) space by using the segmented MRI data as a reference.

### Modelling intrastriatal connectivity using complex networks

In contrast to other models of the striatum, we leverage a graph network modelling the connections of neurons under the hypothesis the graph properties are linked to biological activities. Thus, four main graph indices characterise the structural connectivity of the network, i.e. degree distribution (number of connections of each neuron), connection efficacy (minimal distance between nodes), clustering coefficients (numbers of locally interconnected triplets) and the degree of betweenness-centrality, i.e. the number of neurons serving as high-density hubs. The knowledge of the topological structure of the network plays an important role in emergent neural activity and functionality^42,56^. Furthermore, knowledge of structural details allows us to investigate the mechanisms involved in neural functionality or dysfunctionality. Network structural properties, in turn, can be identified using network statistical measures as listed above (i.e. degree distribution average path length, clustering coefficients, centrality^38,49,57^). In our case, the positions of nodes were defined by the data source analysis. The following section will describe how these nodes are connected to each other.

#### Construction of complex network using the structural connectivity

Initially, data source analysis defined a network consisting of 1,995 nodes. In this network, we assume that the majority of the nodes (95%) represent MSN neurons, and the remaining 5% are interneurons, consistent with^58^. The connectivity of the striatum is constructed following the idea of the small-world algorithm^40,49,50,57,59^: initially, each MSN neuron is connected with $k=20$ neurons in the vicinity of 5 $\textrm{mm}$ (local connections). Next, with a small probability *p*, and for each local connection, a new remote neighbour is added (e.g. $p=0.05$). For interneurons, we follow the same structure, using, however, a five times denser interneuron to MSN connectivity (i.e. $k=100$). In this way, the resulting network is highly clustered (like a lattice structure), additionally with a small distance between nodes (like random networks).

Unlike traditionally used lattice connectivity models or all-to-all models, small-world structures better represent the physiological networks as a result of two main characteristics^44,50,60–64^: they are highly clustered, and typically show short path lengths^38,49,59,65^, enhancing in this way signal or rhythm propagation within the network, and the synchronisability of the network. We constructed the striatum network phenomenologically (no exact knowledge of the individual neuron connectivity in the striatum is known). Nevertheless, the choices of local connections per node *k* and the probability of random remote connections *p* in the model are successfully chosen so that the network shows higher values of clustering coefficients than a random network (where the clustering coefficients have very small values). In this sense, our choice produces more realistic connectivity features (compared, e.g., to fully connected or randomly connected models), which are closer to biological (brain) systems.

The resulting striatal network is represented as graph $G=(V,\, E)$, where *V* is the set of nodes and *E* represents the set of edges, i.e. connections. The nodes of the structural network are defined as points in three-dimensional space, as a single neuron with spatial coordinates. The connectivity can be represented with the adjacency (or connectivity) matrix *A*: if two neurons at positions ${\textbf {x}}=(x_1,y_1,z_1)$ and ${\textbf {y}}=(x_2,y_2,z_2)$ are linked then $A({\textbf {x}},{\textbf {y}})=1$, otherwise $A({\textbf {x}},{\textbf {y}})=0$. In the next section, we provide tools that allow us to extract the connectivity properties of the striatal network.

#### Network measures characterising striatal connectivity

Network measures^59^ reveal important properties of the structure, e.g. the expected number of connections for each node of the network, the centrality of nodes (importance of nodes), the average shortest path (expected number of steps between any two nodes), and, importantly, the detection of communities (or modules) and how these organise the network by creating dynamical patterns^42,56^. A network measure can express a specific property of a node (e.g. the clustering property of one node) and appears as an individual property of the *i*th node, while the resulting distribution over the whole network defines the global-macroscopic description (e.g. the statistical distribution of clustering).

#### Degree distribution

The degree of a node *i* refers to the number of links connected to it^59^. In directed networks, a node has both an in-degree and out-degree, numbering in-coming and out-going edges, respectively. A high degree of connectivity (increased numbers of links) of the *i*th node defines the importance of a node in the network. The degree distribution *P*(*k*) defines the probability of a randomly selected node having a specific degree *k*.

#### Path lengths, efficacy and clustering coefficient

The minimum number of steps between two nodes in the network (in the case of binary networks such as the one described here) defines the shortest path length $d_{i\rightarrow {j}}$ between node *i* and *j*. Averaging over the set of all shortest paths, we obtain the mean path length of the network^59^:

1 $$\begin{aligned} \overline{m}=\frac{\sum _{i,j} d_{i\rightarrow {j}}}{N(N-1)}. \end{aligned}$$

The mean path length shows the ability of the network to spread information (signal activity) between any two nodes. A low mean shortest path length $\overline{m}$ highlights that any two randomly chosen nodes can interchange information very fast.

If no pathway exists between node *i* and *j* then $d_{i\rightarrow j}=\infty$, and this pair of nodes is excluded from Eq. (1). A similar measure reflecting pathway length, which, however, avoids divisions by 0, is the global efficacy $\overline{l}$^59^:

2 $$\begin{aligned} \overline{l}=\frac{\sum _{i,j} \frac{1}{d_{i\rightarrow {j}}}}{N(N-1)}. \end{aligned}$$

now $d_{i\rightarrow j}=\infty \implies 1/d_{i\rightarrow j}=0$. The inverse global efficacy $1/\overline{l}$ is thus reflecting the mean shortest path.

Another measure that characterises the local connectivity is the clustering coefficient. It measures the proportion of triangle loops that exist in a node, expressing a feedback mechanism that enhances the rhythm generation. Specifically, the clustering coefficient of a node *i* is defined as ratio:

3 $$\begin{aligned} c(i) =\frac{\sum _{jk} a_{ij}a_{jk}a_{ki}}{k_i(k_i-1)}. \end{aligned}$$

The higher the number of triangles (that exist) with respect to the *i*th node, the higher the clustering coefficient.

#### Betweenness centrality

Another   significant measure which quantifies the importance of a node is ‘betweenness centrality’. The term ‘centrality’ is related to the degree of influence which this particular node exerts within the network. ‘Betweenness centrality’ thus measures the amount of influence, which a node has with respect to the total information flow in the network. Important nodes that connect different subgraphs in the network (i.e., act as a bridge) show high betweenness centrality. The betweenness centrality *BC*(*i*) of the *i*th node is mathematically defined as the fraction of all shortest paths in the network that pass through the node, that is,

4 $$\begin{aligned} BC(i)=\sum _{j\ne i \ne k}(g_{jk} (i))/g_{jk}\,, \end{aligned}$$

where $g_{jk} (i)$ is the number of shortest paths from *j* to *k* passing over *i*, and $g_{jk}$ is the number of shortest paths between nodes *j* and *k*. Bridging nodes that connect different subsets of the network often have a high betweenness centrality. Higher values of *BC*(*i*) indicate that the node acts as a central hub. The importance of these hubs is also highlighted pathophysiologically as such hubs are ideally suited as targets of therapeutic intervention, i.e. for DBS^38^.

### Detection of communities and modularity

Networks characteristically contain subsets (subgraphs) that have dense internal connectivity (connectivity among nodes in the subset) and sparse connections to other subgraphs^65^. The partition of the network into densely connected subgraphs (or communities) plays a significant role in information processing within the network, and it is also related to different biological functions of the area (e.g. striatum)^66^. Assigning and allocating these densely connected communities to brain structures allows the construction of a modular view of the dynamics of the network^67,68^.

The modality index identifies such densely connected communities. The modality index^65^ assigns a community number $s_i$ to each node. For example, if there are two communities, then $s_i=\pm 1$. Here, we seek the best network partition in order to maximise the modularity function *Q*:

5 $$\begin{aligned} Q=\frac{1}{4m}{s}^TBs \end{aligned}$$

where $m=1/2 \sum k_{ij}$ is the total number of edges in the network, and $B_{ij}=A_{ij}-k_i k_j/2m$ is the resultant modularity matrix, also known as graph Laplacian matrix. In such matrices, the optimisations can be achieved using graph partitioning or spectral partitioning (eigenvalues-eigenvectors decomposition) of *B*^65,69^.

### Modelling and simulation of MSN networks

#### Modelling MSN neurons

The dynamics of each MSN neuron are modelled by current balance equations for the membrane potential:^46^

6 $$\begin{aligned} C\frac{dV_i}{dt}&=-I_{\text {LEAK}}-I_{\text {K}}-I_{\text {Na}}-I_{\text {M}}-I_{\text {syn}}+I_{\text {app}}\, , \end{aligned}$$

7 $$\begin{aligned} \frac{dx_i}{dt}&=(x_{\infty }-x_i)/\tau _{x_i}\, , \end{aligned}$$

where *C* is the membrane capacity, and $V_i$ is the membrane potential of the *i*th neuron. The current balance Eq. (6) contains four membrane currents^46^, the fast sodium and potassium currents $I_{\text {Na}}$ and $I_{\text {K}}$, the leak current $I_{\text {LEAK}}$, and an M-current $I_{\text {M}}$. All ionic currents follow the Hodgkin–Huxley formalism^47^: $I=g_X m^{n_1}_X h^{n_2}_X \cdot (V-E_X)$, where the exponents $n_1, n_2$ represent the number of activation-inactivation channels, respectively, $g_X$ is the maximum conductance of the *X* ion, and $E_X$ stands for the reversal potential for each *X* ion ($X \in \{\text {Na, K}\}$). Specifically, the sodium current has three activation gates and one inactivation gate, that is, $I_{\text {Na}}=g_{\text {Na}} m^3_{\text {Na}} h_{\text {Na}} \cdot (V-E_{\text {Na}})$. The potassium current has the form $I_{\text {K}}=g_{\text {K}} m^4_{\text {K}} \cdot (V-E_{\text {K}})$ (i.e., $n_1 = 4,n_2=0$). Finally, the M-current and leak currents follow $I_{\text {M}}=g_{\text {M}} m_{\text {M}} \cdot (V-E_{\text {K}})$ and $I_{\text {LEAK}}=g_{\text {LEAK}} \cdot (V-E_{\text {LEAK}})$, respectively (see also Table 1).

The variable $x_i$ denotes the gating variables $m_{\text {X}}$ and $h_{\text {X}}$. Following the Hodgkin–Huxley formalism^46,47^, the function $x_{\infty }$ is given by $x_{\infty }=\frac{\alpha _l}{\alpha _l+\beta _l}$ and the time by $\tau _{X}=\frac{1}{\alpha _l+\beta _l}$, $l \in m_{\text {X}}, h_{\text {X}}$. For the sodium current and for the gating variable $m_{\text {X}}$, we obtain

8 $$\begin{aligned} a_m(V)=0.32\frac{V+54}{1-e^{-(V+54)/4)}}, \hspace{0.5cm} b_m(V)=0.28\frac{(V+27)}{e^{(V+27)/5}-1}; \end{aligned}$$

similarly, for the gating variable $h_{\text {X}}$:

9 $$\begin{aligned} a_h(V)=0.128e^{-(V+50)/18}, \hspace{0.5cm} b_h(V)=\frac{4}{1+e^{-(V+27)/5}}. \end{aligned}$$

For the potassium current, with only one activation gating: $m_{\text {k}}$

10 $$\begin{aligned} a_m(V)=0.032\frac{V+52}{1-e^{-(V+52)/5)}}, \hspace{0.5cm} b_m(V)=0.5 {e^{-(V+57)/40}}. \end{aligned}$$

For the M-current, we obtain

11 $$\begin{aligned} a_m(V)=0.032\frac{V+52}{1-e^{-(V+52)/5)}}, \hspace{0.5cm} b_m(V)=0.5 {e^{-(V+57)/40}}. \end{aligned}$$

The current $I_{\text {app}}$ is written as $I_{\text {app}}=I_{0}+I_{\text {DBS}}$ in Eq. (7), where $I_0$ represents a network activation current, describing the dependence of the neuronal activation due to dopamine receptor activation, or due to intensity of cortical-striatal connectivity. The current $I_{\text {DBS}}$ specifically models deep brain stimulation, and it is applied on any element within the reach of the stimulation electrodes (the stimulation is modelled as declining exponentially, see Eq.(12)). The mathematical description is given by the form:

12 $$\begin{aligned} I_{\text {DBS}}=A_{\text {DBS}}e^{-\frac{(x-x_0)^2+(y-y_0)^2+(z-z_0)^2}{\sigma ^2}}H(\sin (2\pi t/T_{\text {DBS}})\cdot (1-H(\sin (2\pi (t+\delta _{\text {DBS}})/T_{\text {DBS}})), \end{aligned}$$

while in the absence of DBS treatment $I_{\text {DBS}}=0$.

The synaptic currents $I_{\text {syn}}$ for MSN neurons can be written as a sum: $I_{\text {syn}}=I_{\text {MSN} \rightarrow \text {MSN}}+I_{\text {FS} \rightarrow \text {MSN}}$, where $I_{\text {MSN} \rightarrow \text {MSN}}$ is the inhibitory synaptic current between MSN neurons, carried by fast-spiking neurons, whose exact description is given in the next section.

#### Modelling Fast Spiking (FS) neurons

The dynamics of each FS neuron are modelled by current balance equations for the membrane potential:^46^:

13 $$\begin{aligned} C\frac{dV_i}{dt}&=-I_{\text {LEAK}}-I_{\text {K}}-I_{\text {Na}}-I_{\text {D}}-I_{\text {syn}}+I_{\text {app}}. \end{aligned}$$

14 $$\begin{aligned} \frac{dx_i}{dt}&=(x_{\infty }-x_i)/\tau _{x_i} , \end{aligned}$$

where *C* is the membrane capacity, and $V_i$ is the membrane potential of the *i*th FS neuron. The current balance Eq. (14) contains four membrane currents^46^:$I_{\text {Na}}=g_{\text {Na}} m^3_{\text {Na},\infty } h_{\text {Na}} (V-E_{\text {Na}})$, $I_{\text {K}}=g_{\text {K}} m^2_{\text {K}} (V-E_{\text {K}})$, $I_{\text {LEAK}}=g_{\text {LEAK}} (V-E_{\text {LEAK}})$, while the fast-activating, slowly inactivating dendritic potassium D-current has the form^46,70^: $I_{\text {D}}=g_{\text {D}} m^3_{\text {D}} h_{\text {D}} (V-E_{\text {K}})$, with three activation gates and one inactivation gate (i.e. $n_1=3, n_2=1$, see also Table 1), thus imposing a delay in firing upon depolarisation. For the sodium current $I_{\text {Na}}$, and for the gating variable *m*, we obtain:

15 $$\begin{aligned} m_{\infty }=\frac{1}{1+e^{-(V+24)/11.5}}, \end{aligned}$$

and for the sodium inactivation

16 $$\begin{aligned} h_{\infty }=\frac{1}{1+e^{-(V+58.3)/6.7}}. \end{aligned}$$

For the potassium $I_{\text {K}}$current, and for the activation *m* variable, we use the equation:

17 $$\begin{aligned} m_{\infty }=\frac{1}{1+e^{-(V+12.4)/6.8}}. \end{aligned}$$

Finally, for the D-current with activation and inactivation variables, we use

18 $$\begin{aligned} m_{\infty }=\frac{1}{1+e^{-(V+50)/20}}, \end{aligned}$$

and for the inactivation

19 $$\begin{aligned} h_{\infty }=\frac{1}{1+e^{-(V+70)/6}}. \end{aligned}$$

**Table 1:** The currents for medium spiny neurons (MSN) and fast-spiking neurons (FS).

Description of current	MSN	FS
$I_{\text {LEAK}}$	$g_{\text {LEAK}}(V_i-E_{\text {LEAK}})$	$g_{\text {LEAK}}(V_i-E_{\text {LEAK}})$
$I_{\text {K}}$	$g_{\text {K}}m_{\text {K}}^4(V_i-E_{\text {K}})$	$g_{\text {K}}m_{\text {K}}^2(V_i-E_{\text {K}})$
$I_{\text {Na}}$	$g_{\text {Na}}m_{\text {Na}}^3h_{\text {Na}}(V_i-E_{\text {Na}})$	$g_{\text {Na}}m_{\text {Na}}^3h_{\text {Na}}(V_i-E_{\text {Na}})$
$I_{\text {M}}$	$g_{\text {M}}m_{M}(V_i-E_{\text {K}})$	–
$I_{\text {D}}$	–	$g_{\text {D}}m_{\text {D}}^3h_{\text {D}}(V_i-E_{\text {K}})$

### Description of the network inhibitory synaptic activity

The coupling between the neurons in eqns. (6) and (13) is described by the synaptic current $I_{\text {syn}}$. Initially, we model the activation of a synapse using the activation variable $s_i$ (for the *i*th neuron), which is given by^71–73^:

20 $$\begin{aligned} \frac{ds_i}{dt} = \alpha (1-s_i)H(V_i)-\beta s_i, \end{aligned}$$

where the function *H*(*V*) is a smooth approximation of the step (Heaviside) function $H_\text {step}$ (i.e. $H_\text {step}(x)=1, x>0$ and $H_\text {step}(x)=0, x<0$.) The variable $s_i$ describes the activation of synapses from the pre-synaptic neuron *i* to the post-synaptic neuron *j*. The form of function *H* is given by:

21 $$\begin{aligned} H(V)=1+\tanh (V/10), \end{aligned}$$

The parameters $\alpha , \beta$ in Eq. (20) are related to the activation and inactivation time scales, respectively, of the inhibitory (GABA-ergic) synaptic connections. In cases of MSN - MSN and MSN - FSI interactions, the activation rates in equation (20) are $\alpha =4, \beta =1/13 \approx 0.08$. Similarly, for FS - MSN and FS - FS interactions, the activation rates in equation (20) are $\alpha =4, \beta =1/11$.

For each *i*th neuron in the network, the total synaptic inhibition which it receives from the pre-synaptic neurons is:

22 $$\begin{aligned} I_{i,\text {GABA}}=g_{\text {X} \text {Y}}(V_i-E_{\text {GABA}})\sum _{j}{A_{ij}s_{j}}, \end{aligned}$$

with $E_{\text {GABA}}=-80mV$. The matrix element $A_{ij}$ has the value 1 or 0, depending on whether neurons *i* and *j* are connected or not. In this way, it resembles the modified Watts and Strogatz (WS) small-world topology^49,57,59,60,63,74–76^. The summation is taken over all presynaptic neurons. The parameter $g_{\text {X}\text {Y}}$ represents the conductance between *X* and *Y* interactions $X, Y \in \{\text {MSN}, \text {FS}\}$.

### Modelling the connectivity within the striatum

The synaptic current for the MSN neurons is given by:

23 $$\begin{aligned} I_{\text {syn}}=I_{\text {MSN} \rightarrow \text {MSN}}+I_{\text {FS} \rightarrow \text {MSN}}. \end{aligned}$$

The current $I_{\text {MSN} \rightarrow \text {MSN}}$ indicates the inhibition between MSN-MSN neurons, while the second term $I_{\text {FS} \rightarrow \text {MSN}}$ represents the interneuronal inhibition. Taken together, the mathematical form of the synaptic current for MSN neurons is:

24 $$\begin{aligned} I_{i,\text {MM}}= g_{\text {MM}}(V_i-E_{\text {GABA}})\sum _{j}{A_{ij}s_{j}}+g_{\text {FM}}(V_i-E_{\text {GABA}})\sum _{j}{A_{ij}s_{j}}, \end{aligned}$$

where, again, the element $A_{ij}$ has the value 1 or 0, depending on whether neurons *i* and *j* are connected or not, while the sum is taken over all presynaptic neurons.

Similarly, for the FS neurons, the synaptic current is analysed as a sum:

25 $$\begin{aligned} I_{\text {syn}}=I_{\text {FS} \rightarrow \text {FS}}+I_{\text {MSN} \rightarrow \text {FS}}. \end{aligned}$$

The current $I_{\text {FS} \rightarrow \text {FS}}$ represents the rare case of FS-FS inhibition, while the second term $I_{\text {MSN} \rightarrow \text {FS}}$ imitates the feedback inhibitory loop of MSN to interneurons. Then, the mathematical form of the synaptic current for each FS neuron is:

26 $$\begin{aligned} I_{i,\text {FS}}= g_{\text {FF}}(V_i-E_{\text {GABA}})\sum _{j}{A_{ij}s_{j}}+g_{\text {MF}}(V_i-E_{\text {GABA}})\sum _{j}{A_{ij}s_{j}}. \end{aligned}$$

### Restoring normal striatal activity by optimising DBS position

As already emphasised, striatal neuronal activity is not only involved in major tasks such as movement control but also in decision-making, reward behaviour and other cognitive/emotional tasks, with behavioural control being driven by the ventral parts of the striatum^1,2^. Under pathological conditions (e.g. obsessive-compulsive disorder), abnormal striatal activity has been reported; decreased dopamine levels in conjunction with aberrant cortico-striatal interactions are thought to lead to reduced striatal network activity^7,27,46^.

In order to quantify striatal activity, we define the mean network activity as a macroscopic variable^77^:

27 $$\begin{aligned} \langle a\rangle _T(t)=\frac{1}{T} \int _{t}^{t+T}{n_A([t, t+T])} dt, \end{aligned}$$

where *N* is the number of neurons in the population, $n_A([t, t+T])$ is the number of spikes (summed over all neurons in the population) that occur between *t* and $t+T$, and where T is a small macroscopic time interval (10 ms). The values obtained were then divided by the number of bins (100); thus, $\langle a\rangle _T$ actually stands for the number of spikes per 0.1 ms bin within the entire population of neurons (2000 neurons). In order to define the mean activity as spikes per neuron in Hz, we ultimately multiply the values by 5 in two steps. In the initial step, the value is multiplied by 10,000 (0.1 ms * 10,000) to obtain a value per second. Subsequently, the value is divided by 2,000 (the number of neurons) in order to obtain the value per neuron (in Hz). Thus, we define the average network activation rate in Hz as $\langle a\rangle _T$. Low values of the macroscopic network rate indicate low striatal output, characteristic of a disturbed and abnormally low dopamine, or low intensity of corticostriatal activity.

Another macroscopic variable that we explore is the mean membrane voltage $\overline{V}$ of neurons in the network; specifically, we define:

28 $$\begin{aligned} \overline{V}_x(t)= \frac{1}{N}\sum _{k=1}^{N}{V_k(t)}. \end{aligned}$$

The mean voltage activity $\overline{V}$ (indirectly related to the local field potential (LFP) in the case of supra-threshold values resulting in spiking) is utilised for the characterisation of synchronised rhythm (through Fourier spectrum) under different states (healthy, abnormal or abnormal plus DBS).

### Optimising DBS parameters using macroscopic quantities of the striatal network

Differences in striatal targeting areas, as well as different intensity and frequency values of the DBS signal, result in differences in distant network activation. In the model, we vary position, stimulation intensity and frequency, resulting in the parameter vector $r=(x_0,y_0,z_0, A_{\text {DBS}},f_{\text {DBS}}) \in \mathbb {R}^{5}$ to estimate optimal DBS outcome; see Eq. (12). The effectiveness of DBS is evaluated using three objective functions. The objective functions defined below indicate the impact of DBS, i.e. the ability of DBS to restore neuronal activity to a healthy state.

#### Optimising with respect to mean network activity

The first objective function is based on the mean network activity $\langle a \rangle _T$ Eq. (27). We define the objective function as the difference between healthy and DBS mean activities, i.e.

29 $$\begin{aligned} \Phi _1(r):=|| \langle a\rangle _{T, X_\text {healthy}}- \ \langle a\rangle _{T,X_\text {DBS}} ||_{L^1} =\int _{a}^{b}{\big |\langle a\rangle _{T, X_\text {healthy}}(t)- \ \langle a\rangle _{T,X_\text {DBS}}(t)\big |}dt, \end{aligned}$$

where *a*, *b* are the times of activation and inactivation of DBS (usually $a=0$ and $b=1$ sec), $r=(x_0,y_0,z_0,A_{\text {DBS}},f_{\text {DBS}}) \in \mathbb {R}^{5}$ is the DBS parameter vector, i.e. the position $(x_0,y_0,z_0)$ of the DBS electrode, the amplitude $A_{\text {DBS}}$ and the frequency *f* of the pulse. The values of the model parameters, $r \in \mathbb {R}^{5}$ were estimated numerically by minimising the residual, i.e.:

30 $$\begin{aligned} \mathop {\mathrm {arg\,min}}\limits _{r \in \mathbb {R}^{5}} \Phi _1(r):= \left\{ r: \min \Phi _1(r),\ r \in \mathbb {R}^{5} \right\} . \end{aligned}$$

Minima of the difference function $\Phi _1$, (similarly  for $\Phi _2$ and $\Phi _3$) , i.e. values of the objective function close to 0 imply $\langle a \rangle_{T, X_\text {healthy}} \approx \langle a \rangle_{T, X_\text {DBS}}$, which it is interpreted as effective DBS action restoration of normal striatal activity.

#### Optimising with respect to network rhythmicity

The second objective function $\Phi _2$ is based on the frequency spectrum produced by the model. For this, we define the Fourier transform of the mean activity $\overline{V}$ i.e.

31 $$\begin{aligned} X_r(f)= \int _{-\infty }^{\infty }{\overline{V}(t)e^{-j f t}dt}, \end{aligned}$$

where *j* is the imaginary unit. The power spectrum is defined as $|P(f)|=|X(f)|^2$. To estimate the effectiveness of DBS, we calculate differences curves based on the following subtraction pairs: $|P_X(f)|-|P_Y(f)|$, where $|P_X|$, $|P_Y|$ are the power spectra of the states, *X*, *Y*, respectively. *X*, *Y* in this case represent the conditions under DBS *X*, and healthy state *Y*.

The objective function $\Phi _2$ is then defined as the area under the curve (AUC,^78^) for each of these difference pairs, i.e.

32 $$\begin{aligned} \Phi _2(r):= \int _{a}^{b} \Big | \ |P_X(f)|-|P_Y(f)|\ \Big | df, \end{aligned}$$

where *a*, *b* are the frequency range (i.e. [a,b]=[0 300]Hz) and $r=(x_0,y_0,z_0,A_{\text {DBS}},f_{\text {DBS}}) \in \mathbb {R}^{5}$ is the DBS parameter vector. Similar to the previous case, i.e. $\Phi _1$, the values of the model parameter, $r \in \mathbb {R}^{5}$ were estimated numerically by minimising the residual, that is,

33 $$\begin{aligned} \mathop {\mathrm {arg\,min}}\limits _{r \in \mathbb {R}^{3}} \Phi _2(r):= \left\{ r: \min \Phi _2(r),\ r \in \mathbb {R}^{5} \right\} . \end{aligned}$$

We can also combine the aforementioned objective functions to obtain a third optimisation scheme that takes into account both macroscopic characteristics (i.e. rate and rhythmicity) of the network activity. We will present this scheme in the next subsection.

#### Optimising using a combination of network rhythmicity and firing rate

We define a combination of objectives functions eqs. (32) and (29):

34 $$\begin{aligned} \Phi _3(r):= \Phi _1(r)+\gamma \cdot \Phi _2(r), \end{aligned}$$

where $\gamma$ is a scaling factor that balances (reduces or increases) the importance of the phase spectrum in the optimisation process. In this instance, $\gamma$ is adapted by iterative optimisation steps with decreasing sizes of $\gamma$. At the chosen value, the objective function $\Phi _3$ combines both $\Phi _1, \Phi _2$ optimally, i.e. considers both the rate and the rhythmicity effect.

The minimisation problem was solved in all cases using a nonlinear least-squares solver (the MATLAB function **lsqnonln**). The step size tolerance was set to $\texttt {tol}(X)=0.001$ and the function tolerance was set to $\texttt {tolF}=0.1$. The maximum number of iterations was set to 100.

**Figure 2:**
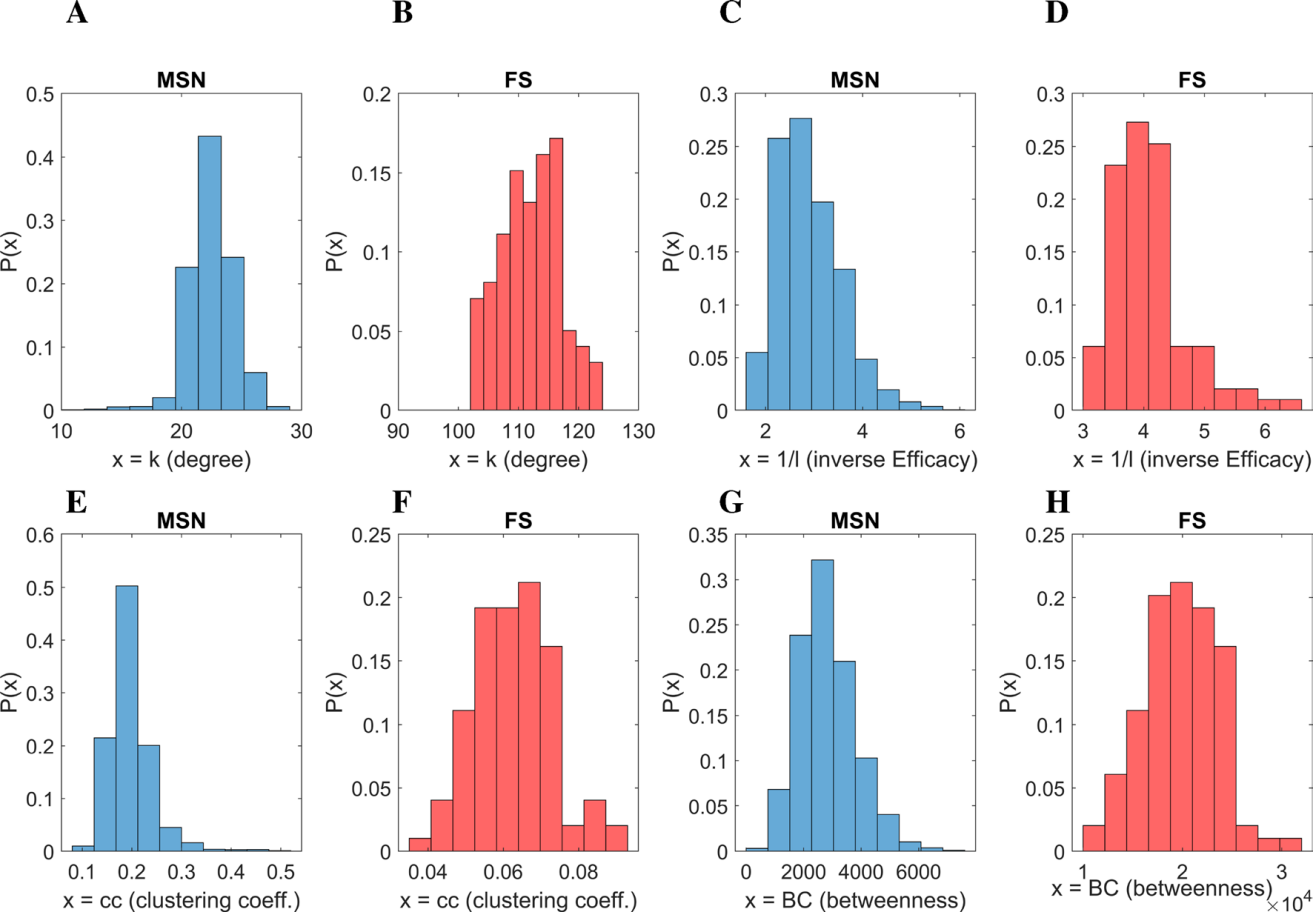
Connectivity properties of the striatal network. Blue histograms stand for the MSN neurons, while red for FS neurons. (**A, B**) Distribution of the number of connections leaving a node (*out-degree distribution*). Clearly, MSN neurons show a relatively symmetrical spread of data with relatively sparse connections (mean 25 connections), unlike fast-spiking GABAergic interneurons (minority; mean 110 connections). (**C, D**) Distribution of *efficacy* (the number of steps as maximal distance between any two neurons, here given as reciprocal value to avoid divisions by 0). (**E, F**) The distribution of *clustering coefficients*, i.e. the number of locally interconnected neuronal triplets. MSN neurons show a relatively high number of triangle loops, see Eq. (3), while FS neurons show a lower number of triangles. (**G, H**) The distribution of *betweenness centrality* (BC), measuring the number of paths passing from a given node. MSN neurons have a lower value of betweenness centrality, while FS neurons show almost 10 times higher values of BC.

**Figure 3:**
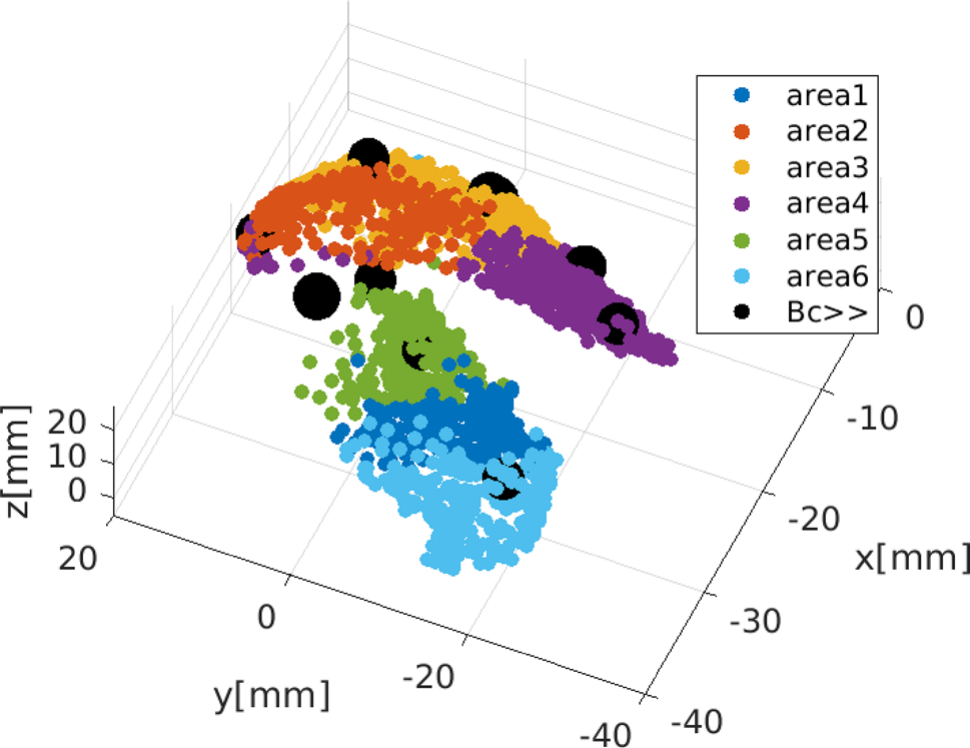
Modularity: Community detection in the striatal network. Communities were extracted using an iterative process of branching groups of neurons fulfilling two criteria: (**a**) dense connectivity among members and (**b**) sparse connectivity to the other communities. This iterative branching stops when an optimum is reached in any branch. In this way, the striatal network is partitioned into separate subgraphs, using a commonly used modularity-index algorithm^69^. Using a boundary condition that at least 180 neurons should be within one community (9 % of the population), in the present model, the algorithm identifies six communities, which remain stable with repetitive (20 times) realisations. As the figure shows, three communities are located in the caudate nucleus (top, red, yellow and violet hues) and three in the putamen (bottom, blue and green hues). The ten nodes with the highest betweenness-centrality (hubs, black circles) are equally distributed between the caudate nucleus (n = 5) and the putamen (n = 5).

### Sensitivity analysis

The last part of our theoretical analysis (Sensitivity Analysis) is further investigating the model parameters. Specifically, the aim is to examine the significance or the sensitivity of the emergent network behaviour with respect to vital parameters such as the intensity of cortico-striatal connectivity and the connectivity conductance between MSN neurons. Using Sobol’ indices as order parameters, we are partitioning the variance of the output into fractions according to the parameter’s input contribution. The Sobol’ indices lie in the interval [0, 1]. A Sobol’ index close to 1 reflects a more significant influence of the parameter on the model’s response.

For the global sensitivity analysis, i.e., to quantify the effects of the input random variables in the variance of the response of the model, we use Sobol’ indices^79^, based on functional decomposition applied to the variance. The implementation and use of the method are straightforward. In the system of interest, we consider $\textbf{X}$ as a random input vector following a certain probability density function $f_\textbf{X}$, and $\textbf{Y}$ as the response of this system.

The total variance of a model’s response is denoted by $\textrm{var}(\textbf{Y})$, and the conditional variances, which consider the contribution of one or more parameters, are denoted by $\textrm{var}[\mathbb {E}(\textbf{Y}|\textbf{X}_k)]$, where $\textbf{X}_k$ is the input vector with *k* the parameters, with $k\in \mathbb {N}$. From Sobol’ decomposition and its orthogonality, we obtain the total variance of $\textbf{Y}$ as the sum of the conditional variances, i.e.,

35 $$\begin{aligned} \textrm{var}(\textbf{Y}) = \sum \limits _{k} \textrm{var}[\mathbb {E}(\textbf{Y}|\textbf{X}_k)], \end{aligned}$$

where $\mathbb {E}$ is the expectation. In this sense, Sobol’ indices are defined by

36 $$\begin{aligned} S_k = \frac{\textrm{var}[\mathbb {E}(\textbf{Y}|\textbf{X}_k)]}{\textrm{var}(\textbf{Y})}, \end{aligned}$$

such that their total sum equals one. Therefore, a Sobol’ index can have a value within the interval [0,1]. The closer the sensitivity index of a parameter approaches the value 1, the greater its influence on the response of the model.

The aim is to examine the significance of a particular variable by measuring the proportion of variance in the Quantity of Interest (QoI) for which it is responsible. For this, we compute the first-order Sobol’ index, which quantifies the share of variance in the output due to the examined parameter. In addition, the higher-order index quantification considers the interaction of all studied variables.

To compute Sobol’ indices, we choose the Polynomial Chaos Expansion (PCE), which has proven to be very efficient in dealing with uncertainties. The PCE method is an efficient alternative to the Monte-Carlo methods, being much faster in obtaining similar results, as long as the number of uncertain variables is less than 20, see, e.g.,^80^. PCE can significantly decrease the number of simulations while providing an accurate approximation of the model’s response.

The PCE method uses an approximation of a system’s random model response $\textbf{Y}$, assuming that $\textbf{Y}$ has finite variance. The representation of $\textbf{Y}$ can be given by

37 $$\begin{aligned} \textbf{Y} = \sum \limits _{n=0}^{N_p-1}c_n\Psi _n(\textbf{X}), \end{aligned}$$

with $N_p$ as the number of expansion factors, $c_n$ as the coefficients, and $\Psi _n$ as a multidimensional generalised PC basis defined in a Hilbert space $L^2(\textbf{X},f_\textbf{X})$. The coefficients are computed following

38 $$\begin{aligned} c_n = \frac{\langle \textbf{Y}, \Psi _n(\textbf{X})\rangle }{\langle \Psi _n,\Psi _n\rangle }, \quad \forall \, n=0,1,..., N_p-1. \end{aligned}$$

The equation above is obtained by taking the inner product of Eq. (37) and $\Psi _n$ and using the orthogonality of the basis. The coefficients are calculated with a pseudo-spectral projection method.

In particular, since we are using the uniform distribution $f_\textbf{X}$ of the parameters, $\Psi _n$ is based on Legendre polynomials, which are orthogonal with respect to the uniform distribution. These polynomials are obtained with a three-term recurrence formula,

39 $$\begin{aligned} P_0(x)= & {} 1, \end{aligned}$$

40 $$\begin{aligned} P_1(x)= & {} x,\end{aligned}$$

41 $$\begin{aligned} (n+1)P_{n+1}(x)= & {} (2n+1)xP_n(x)-nP_{n-1}(x), \end{aligned}$$

where $P_n(x)$, $n\in \mathbb {N}$, denotes the Legendre polynomials.

Once the simulations are complete, both an approximation of the model output and the calculated statistics of the QoIs based on the PCE are available.

Many statistical measures can then be obtained directly from the PCE representation, such as the mean and variance of the model response. The Sobol’ indices can also be computed based on the polynomial chaos decomposition of the model^81^ and are called PC-based Sobol’ indices.

## Results

In this section, we present firstly the striatum network’s connectivity properties. Then, we proceed with the optimisation result and the sensitivity analysis.

### Striatum network properties

Initially, data source analysis defined a network consisting of 1,995 nodes. The degree distribution is depicted in Fig. 2A, B. The probability density is depicted separately for the medium-spiny (MSN) and fast-spiking (FS) neurons. Clearly, MSN neurons show a relatively symmetrical spread of data with relatively sparse connections (mean 25 connections), while fast-spiking GABAergic interneurons depict a mean of 110 connections.

The mean shortest path length was found to be approximately 4, i.e., any two randomly chosen neurons can interchange information very fast, passing through very few intermediate nodes. Similarly, the distribution of inverse efficacy $1/\overline{l}$ is depicted in Fig. 2C, D with the mean value computed $1/\overline{l} \approx 3$ and 4 for the FS. Figure 2E, F depicts the distribution of clustering coefficients. The mean clustering coefficient for the MSN neurons is computed as $\overline{c}=0.22$, while for the FS neurons, the clustering coefficients are one magnitude less. Finally, Fig. 2G, H depicts the distribution of betweenness centrality *BC* for the MSN and FS, respectively, of the network. As becomes evident, the MSN neurons have a lower value of betweenness centrality, while FS neurons show almost 10 times higher BC. In Fig. 3, black-filled circles depict the spatial localisation of these central nodes in the network.

### Communities and modularity

Figure 3 shows the communities for the striatal network as determined by the optimisation (maximisation) of the *Q* function. The detected communities emerge as positioned within the boundaries of the brain nuclei; i.e., they follow striatal anatomy, which is likely related to functional somatotopy in the sense that^66^: ‘the limbic loop connecting the ventral striatum with the ventromedial prefrontal cortex (vmPFC) has been implicated in motivational and emotional processing, whereas the associative and sensorimotor networks regulate different forms of behaviours in instrumental behaviour processing. The associative network connecting the dorsomedial striatum with the dorsolateral prefrontal cortex (dlPFC) mainly contributes to goal-directed behaviours, while the sensorimotor network projecting from the dorsolateral striatum to the sensorimotor cortex is mainly responsible for the habitual control behaviours in instrumental learning. In our case, six communities emerged from the simulation as populations with 294, 473, 189, 399, 290, and 330 members, respectively. Interestingly, also in clinical and anatomical literature, the striatum, or more precisely the caudate and putamen, can be functionally subdivided into dorsomedial and ventrolateral parts (communities) on the one hand^66^, and anterior and posterior areas on the other^66^^82^.

### Simulating the healthy state

**Figure 4:**
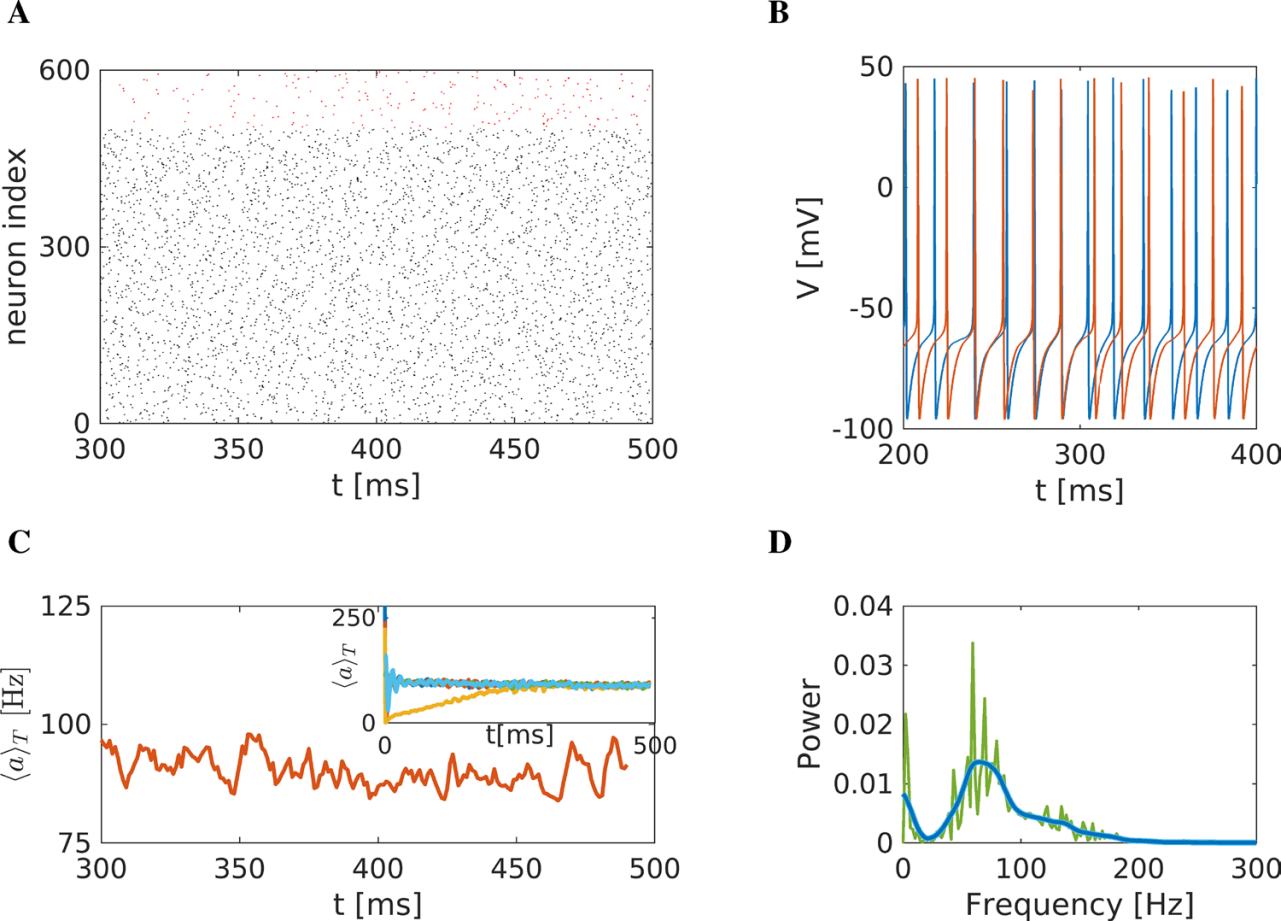
Representation of the striatal network dynamics under healthy conditions (**A**) Raster plot representation. Black dots represent MSN-activated neurons (i.e. action potentials defined as transients passing $V=-15$ mV to positive values), and red dots activated FS neurons (indexed from 500 to 600) against time (in ms) and space (i.e. index of neuron of the nucleus). (**B**) Time series of two representative medium spiny neurons (MSN) of the striatum. (**C**) Mean activity of the striatal network fluctuates around 90-100 Hz. The inset depicts simulations of the model with different initial conditions. Independently of the initial conditions, the network shows a steady state behaviour. (**D**) Power spectrum of the mean membrane potential changes; see Eq. (28), showing high activation in the $\gamma$ band, i.e. at frequencies $>35$ Hz. Green: High-resolution spectrum with a partitioning of 0.01 Hz. Blue: Smoothened curve using Gaussian function smoothing. In the high-resolution spectrum, the three main peaks are found at 59, 69 and 79 Hz, while in the smoothened one, the peak is at 65 Hz.

Parameters were tuned to simulate normal (healthy) conditions. The main characteristic we aimed to achieve is the emergence of $\gamma$ rhythm, as it is also observed in clinical studies^83,84^. The current $I_0$ (expressing dopamine functionality and/or cortical excitation) was set to 5 $\mu$A/$\text {cm}^2$. Figure 4 depicts the overall neuronal activity of the striatum under these normal conditions.

The raster plot (Fig. 4A) shows the activity of 500 randomly chosen neurons and 99 FS neurons (red colour); the network under these conditions apparently is not very much synchronised. Two representative MSN neurons are shown in Fig. 4B. These neurons exhibit spiking activity with variable periods (i.e. non-constant period between two spikes), and some neurons appear to show brief intervals of synchronised activity, preceded and followed by non-synchronous firing. Such synchrony could either be due to transient common activation via network inputs (e.g. inhibition of fast-spiking neuron), or it could actually occur by chance with this tonic firing at a relatively high frequency. The mean network activity $\langle a\rangle _T$, i.e. Eq. (27) as the macroscopic variable quantifying striatal activity, is depicted in Fig. 4C. It fluctuates around its mean value of 90 Hz (action potentials/neuron within 1 second). Additionally, the inset depicts simulations of the model with different initial conditions. Independently of the initial conditions, the network shows a steady state behaviour.The Fourier analysis of the mean membrane potential $\overline{V}$, in turn, is depicted in Fig. 4D. The main characteristic of the power spectrum is a broad interval of $\gamma$ band activity ($f>35$ Hz) with a main peak at $\approx 65$ Hz and a secondary smaller peak at $\approx 3-5$ Hz (blue curve in Fig. 4D).

**Figure 5:**
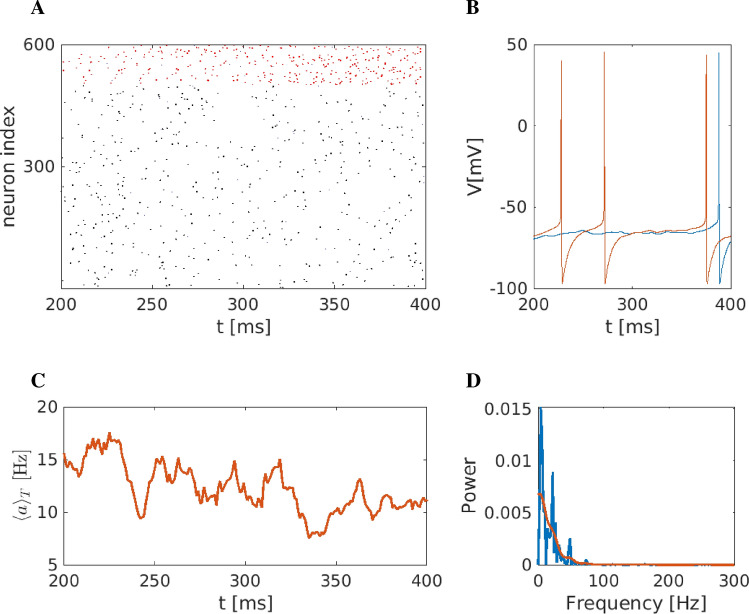
Representation of the striatal network dynamics under abnormal state at low external activation (**A**) Raster plot representation. Black dots represent activated MSN neurons (i.e. action potentials defined as transients passing $V=-15$ mV to positive values), and red dots show the activated FS neurons (indexed from 500 to 600) against time (in ms) and space (i.e. index of neuron of the nucleus). Compared to the healthy state, the raster plot shows very sparse activity. (**B**) Time series of two representative medium spiny neurons (MSN) of the striatum. (**C**) The mean activity rate of the striatal network shows abnormally low activity compared to a healthy state. (**D**) Power spectrum of the mean membrane potential changes; see Eq. (28). The gamma peak formerly present under normal conditions has now all but vanished, and what remains are minute power peaks at about 4, 22, and 55 Hz. Please note different ordinate scaling.

### Simulating abnormal low cortico-striatal activity

Consistent with the hypothesis that both OCD and depression are associated with a reduction in dopaminergic (tegmental) and glutamatergic (cortical) activity^11,21–23^, we model a low excitation tone from the cortex to the striatum^21^ to mimic these pathologies, which we refer to in this paper as “abnormal condition”. For this purpose, we reduce the excitation current by approximately three-fold from $I_0=5$ $\mu$A/$\text {cm}^2$ to $I_0=1.5$ $\mu$A/$\text {cm}^2$, which significantly changes the behaviour of the system. Figure 5 shows the overall network dynamics under these abnormal conditions.

The raster plot in Fig. 5A shows the activity of 500 randomly chosen neurons and 99 FS neurons (red colour); comparing this to the raster plot under normal conditions of Fig. 4A, the main emergent characteristic is weak network activity, which is sparse and strongly decreased compared to healthy activity. Two representative neurons are shown in Fig. 5B. The membrane potential traces confirm the abnormally low activity, with long intervals of neuronal silence (no spiking activity); in effect, neuronal activity in this example is reduced four-fold. The mean network activity $\langle a\rangle _T$, i.e. Eq. (27), is depicted in Fig. 5C. This mean network activity also reflects its reduced activity, which now fluctuates around 15 Hz (15 action potentials per neuron within 1 second); again, this amounts to approximately six-fold reduction. Lastly, the Fourier analysis of the mean membrane potential $\overline{V}$ shows the most important change: While activity is reduced by approximately the degree of the input (three-fold input reduction and four to six-fold activity reduction), the rhythmicity is affected far more strongly. In essence, the gamma rhythm is reduced to approx. 78% of the healthy state (the area under the spectrum curve between [30, 80] Hz of Supplementary Fig. S1 is reduced from 0.9 of the total area in the healthy state to 0.2 of the total area in the abnormal state); see Fig. 5D and Supplementary Fig. S1. The power spectrum is thus now shifted towards low frequencies with a main peak $\approx 4$ Hz indicating a $\theta$ rhythm, a secondary peak at 22 Hz, i.e. $\beta$ band, and a minute peak at around 55 Hz.

We were thus interested in whether modelling could reveal a critical level of input at which the gamma peak would start to disappear and thus conducted modelling with different low levels of input current. As a result, we could show that the critical level appears to be at around $I_0 \approx 3$ $\mu$A/$\text {cm}^2$, see Fig.S1 of the supplementary material, where the intensity of $\gamma$ band activity is computed with respect to the corticostriatal current $I_0$.

**Figure 6:**
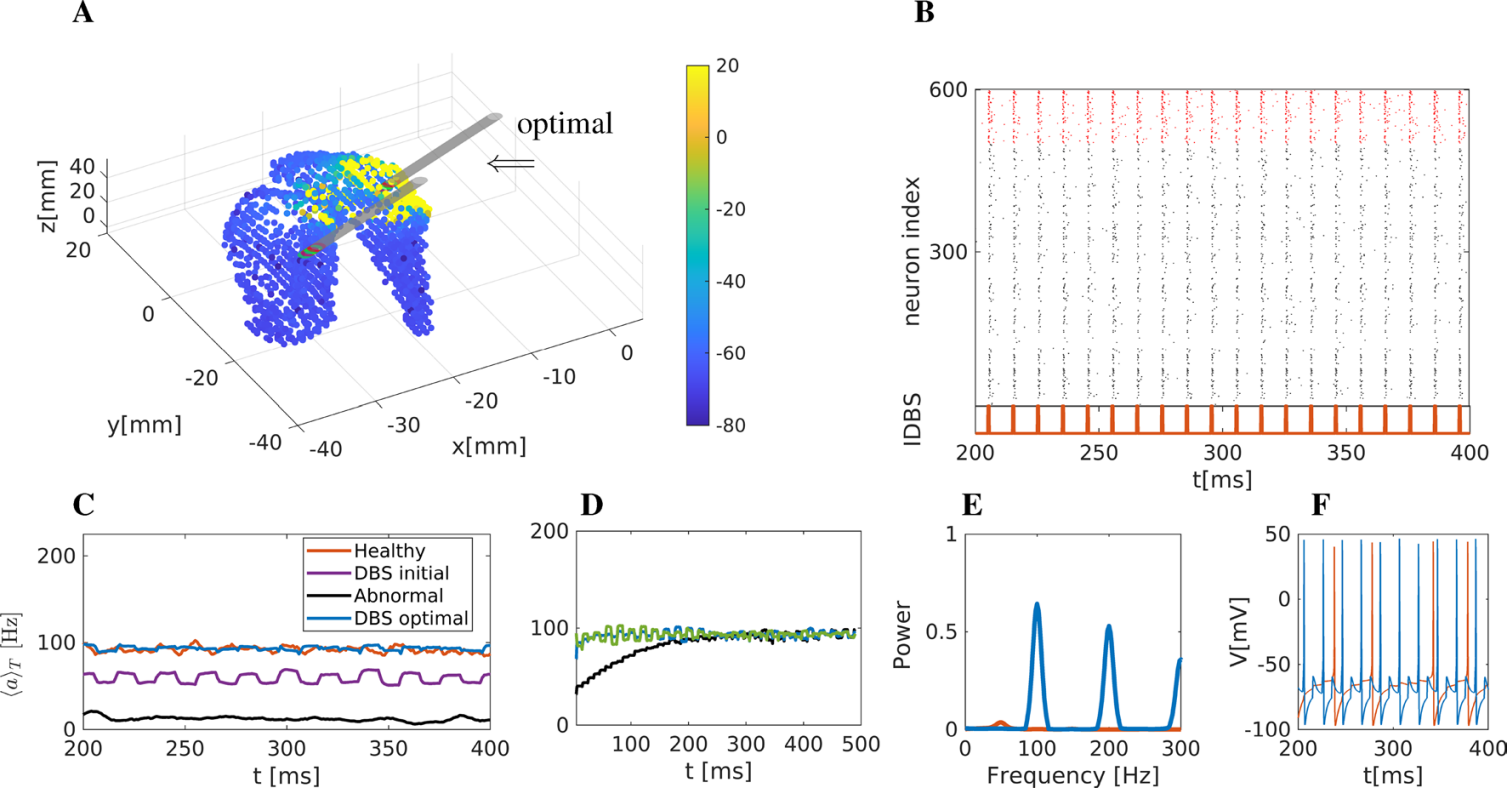
Optimisation of DBS activity on striatal network based on mean activity only. (**A**) Snapshot of striatal activity during DBS. The colour code depicts the predicted mean membrane potential of neurons affected by the stimulation (in mV). Two electrodes are included: one corresponds to the optimal position, marked with an arrow, while the second is the initial position at the beginning of the optimisation process. (**B**) Raster plot representation. Black dots represent activated MSN neurons. Due to the stimulation, the activity is highly synchronised. Red dots show activated FS neurons (indexed from 500 to 600). The DBS current is also depicted below the raster plot. (**C**) Mean network activation of the striatal network. The thick blue line (mean network activity) results from stimulation at an optimal DBS position. For comparison, we also show mean activities under healthy, pathological, and DBS conditions with stimulation in the initial, non-optimised position. (**D**) We illustrate the robustness of the optimisation results based on the network dynamics: we repeat the simulation for different initial conditions using the optimisation values for the model parameters. After a short period, the mean activity converges to the value $\langle a\rangle _T \approx 100$ Hz, very close to the healthy one. (**E**) Power spectrum of the mean membrane activity $\overline{V}.$ The spectrum of the healthy state is shown in red for comparison. (**F**) Two representative neurons were simulated using a stimulation set at optimal DBS conditions.

### Simulating abnormal state using optimal DBS parameters

In this section, we present the optimisation results for network dynamics using three approaches based on the minimisation of the differences (a) of mean network activity in the entire striatum under healthy and abnormal conditions, i.e. when minimising the objective function $\Phi _1$ Eq. (29) only, (b) of network rhythmicity (as determined by the spectrum of the mean membrane activity on the network which is correlated to local field potentials (LFP)) i.e. when minimising the objective function $\Phi _2$ Eq. (32), (c) a combination of mean network activity, and network rhythmicity of the striatal network, i.e. when minimising the objective function $\Phi _3$ Eq. (34). To model the influence of DBS on a network in an abnormal state, the network structure and the model parameters were kept at the abnormal striatum state. Based on the deviation from the healthy state, we propose a DBS protocol that provides the therapeutic pattern for abnormal striatal network activity.

#### Optimised DBS parameters with respect to the mean network activity

The optimal values for the position, frequency and amplitude of DBS using the objective function $\Phi _1$, were found to be $r=(x_0, y_0, z_0, A_{\text {DBS}}, f_{\text {DBS}}) = (-11.39, 4.21, 15.11, 195.02, 99.73)$.

The optimal position for DBS, together with a network snapshot, is depicted in Fig. 6A. The raster plot (Fig. 6B) shows the strongly synchronised activity of the network due to DBS. The mean network activity is depicted in Fig. 6C, jointly with healthy, pathological, and DBS at the initial position, for comparison reasons. Clearly, the mean network activity resulting from optimised (blue line) DBS is in very good agreement with the mean network activity found in the healthy state (red one). In Fig. 6D, we repeat simulations for different initial conditions using the optimization values for the model’s parameters. After a short period, the mean activity converges to the value $\langle a\rangle _T \approx 100$ Hz very close to the healthy one, showing the robustness of the optimisation results.

The spectrum (Fourier analysis) of the mean activity $\overline{V}$ is shown in Fig. 6E. The power spectrum shows high peaks at $\approx 100$ Hz and $\approx 200$ Hz as a result of high-frequency DBS. Finally, firing patterns of two representative neurons are depicted in Fig. 6F. The neurons show activity restored close to healthy conditions.

**Figure 7:**
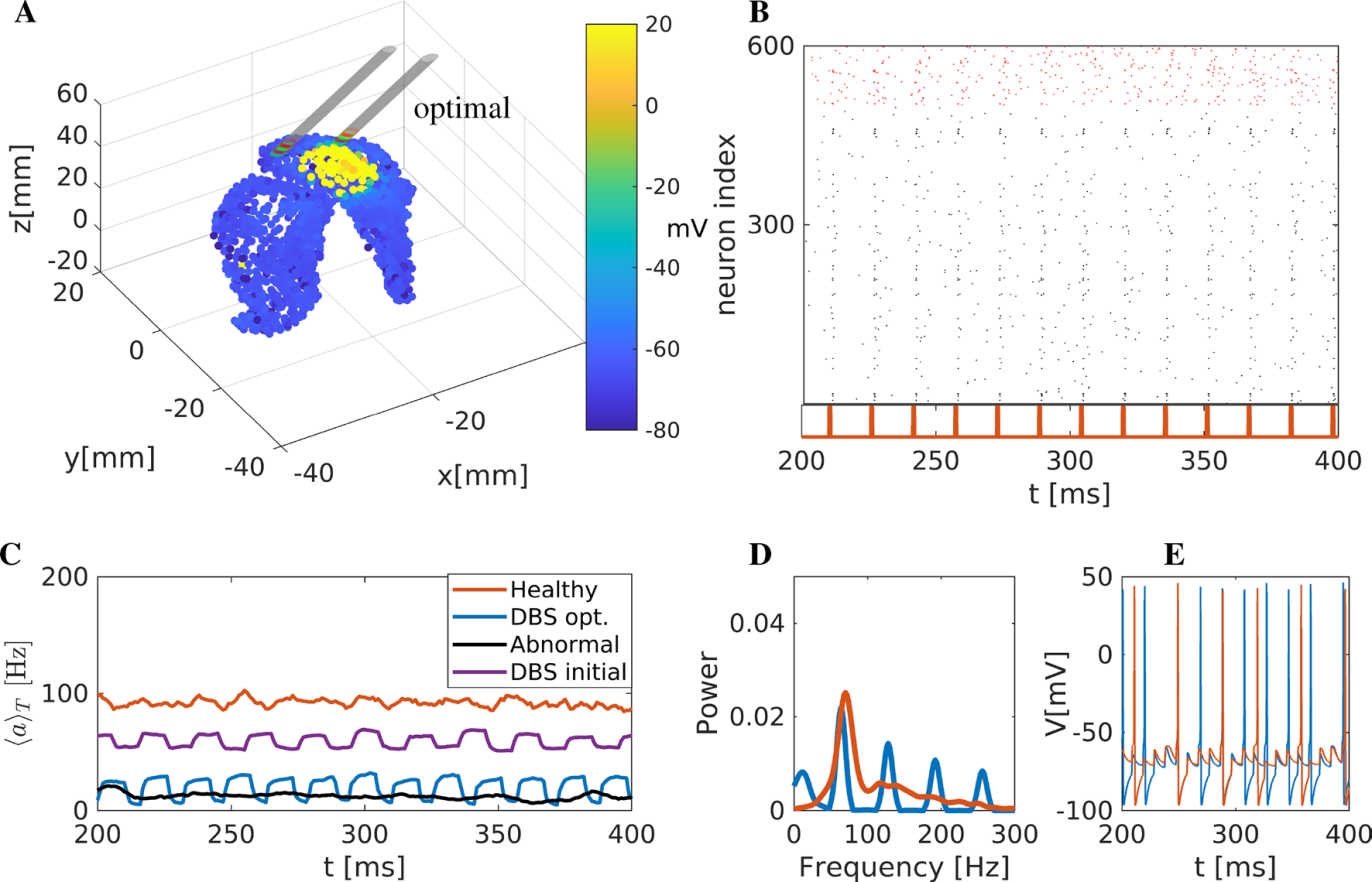
Optimisation of DBS with respect to the network rhythmicity (**A**) Snapshot of the striatum activity during DBS. Colour coding is according to the membrane potential (in mV). Two electrodes are included; one corresponds to the optimal position, marked with an arrow, while the second marks the initial position. (**B**) Raster plot representation. Black dots represent activated MSN neurons, and the activity is synchronised. Red dots show activated FS neurons (indexed from 500 to 600). The DBS current is also depicted below the raster plot. (**C**) Firing rates of the striatum network. Blue thick line (firing rate) resulting from the optimal DBS position. For comparison reasons, we include the healthy, pathological and DBS on a refereed position. (**D**) Power spectrum of the mean membrane activity $\overline{V}$. (**E**) Two representative neurons were simulated using optimal DBS conditions.

**Figure 8:**
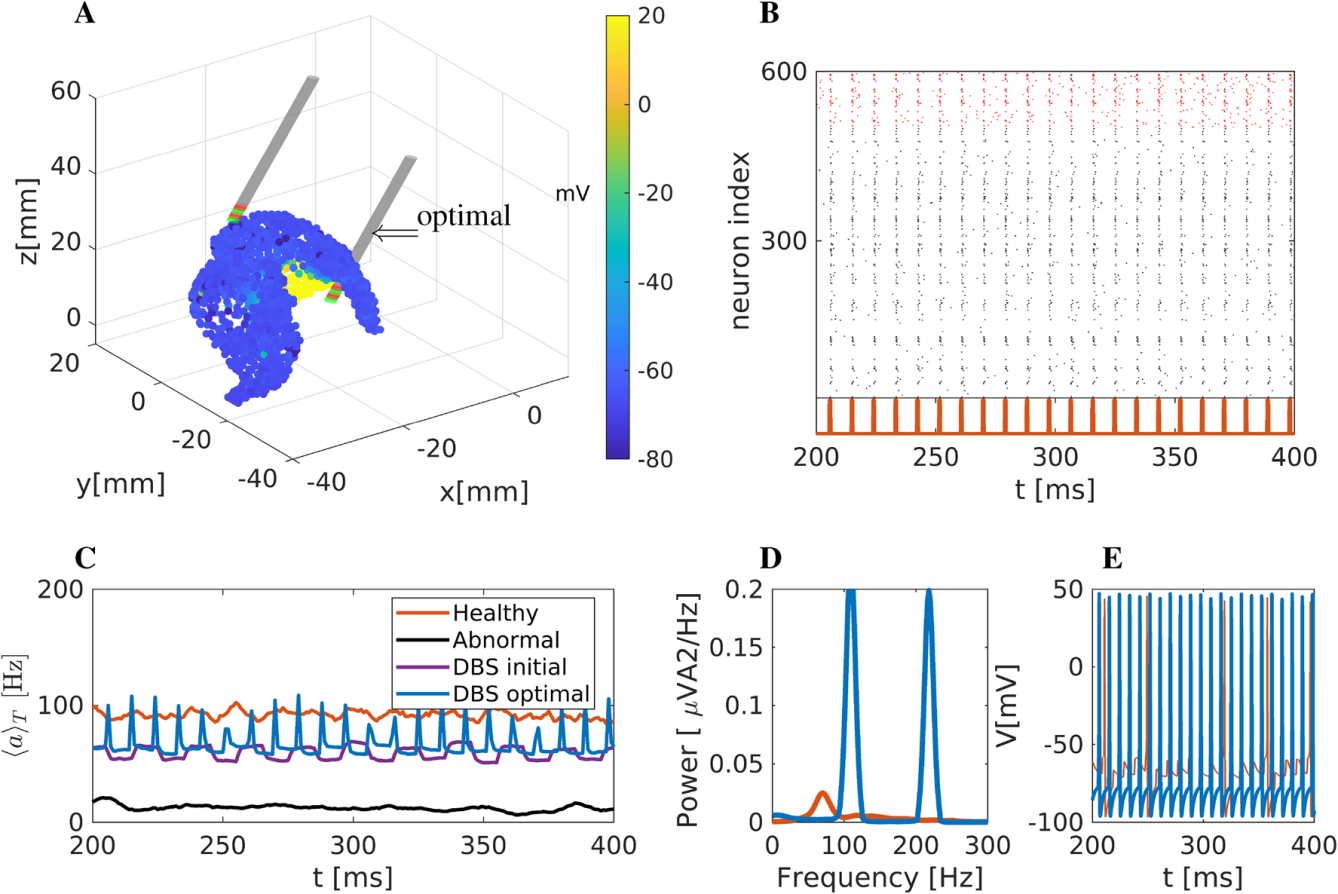
Improved optimisation of DBS with respect to mean network activity and additionally phase and firing rate of the striatal network. (**A**) Snapshot of striatal activity during DBS. The colour code depicts the predicted mean membrane potential of neurons affected by the stimulation (in mV). Two electrodes are included: one corresponds to the optimal position, marked with an arrow, while the second is the initial position at the beginning of the optimisation process. (**B**) Raster plot representation. Black dots represent activated MSN neurons, while red dots represent the activated FS neurons. Due to the stimulation (depicted below the raster plot), the activity is highly synchronised. (**C**) Mean network activation of the striatal network. Blue thick line (mean network activity) results from stimulation at the optimal DBS position. For comparison, we also show mean activities under healthy, pathological, and DBS conditions with stimulation in the initial, non-optimised position. (**D**) Power spectrum of the mean membrane activity $\overline{V}$. (**E**) Two representative neurons were simulated using a stimulation set at optimal DBS conditions.

**Figure 9:**
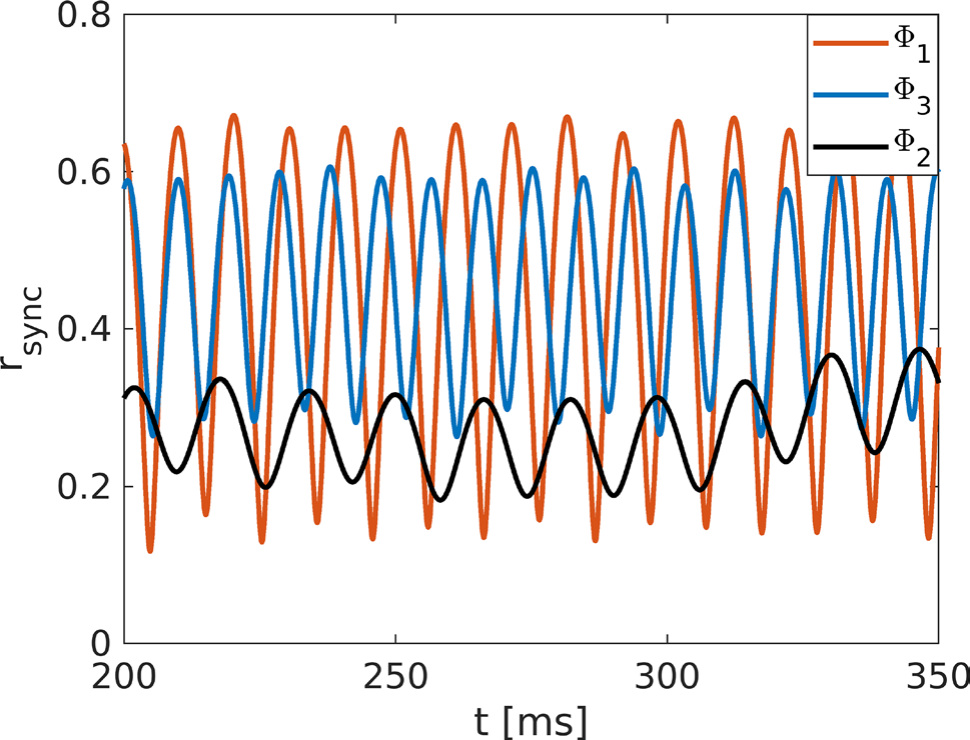
Synchronisation of the network dynamics. In this illustration, we present the synchronisation index for the striatal network under three distinct conditions: Firstly, the dynamics produced from the optimised values of $\Phi _1$ (red curve, optimising the mean activation network activity only) are used, then those produced from $\Phi _2$ (black curve, optimising only the power spectrum), and finally those produced from $\Phi _3$ (blue curve, optimising both). The application of the first optimisation strategy using $\Phi _1$results in a strong synchronisation with a maximum value of $r_{\text {sync, max}} = 0.67$. The second approach, utilising the optimised values of $\Phi _2$, results in a markedly reduced level of synchronisation with $r_{\text {sync, max}} = 0.36$. Finally, for the third optimisation method, employing $\Phi _3$, yields $r_{\text {sync, max}} = 0.58$.

**Figure 10:**
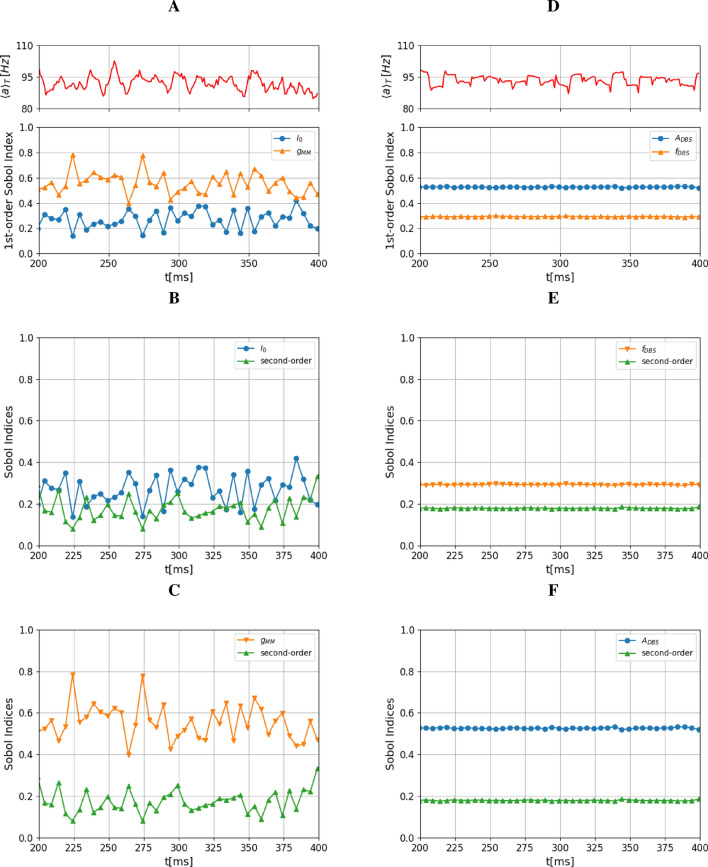
First- and higher-order Sobol’ indices over time in the interval [200 ms, 400 ms]. (**A**) On top, the firing rate is shown. Below, the first-order Sobol’ indices of the two parameters, cortical excitation current ($I_0$) and conductance between MSN neurons ($g_{MM}$), are displayed. The conductance between MSN neurons ($g_{MM}$) is dominant in the firing rate. (**B**) Individual contribution of $I_0$ and the second order Sobol” index. The latter indicates the joint contribution of $I_0$ and $g_{MM}$ to the output variance due to their interactions. (**C**) This figure shows the individual contribution of $g_{MM}$ and the second order index. The range of the second-order Sobol’ index suggests that the interactions between these parameters contribute to the output variance, but not as much as the individual contributions of $I_0$ and $g_{MM}$ separately. Thus, the conductance of MSN neurons predominantly influences the firing rate. (**D**) This figure shows the first-order Sobol’ indices of the parameters, DBS amplitude $A_{\text {DBS}}$ and DBS frequency $f_{\text {DBS}}$ during DBS condition. The amplitude of the DBS current $A_DBS$ has a major influence on the firing rate compared to the frequency of the DBS pulse. (**E**) The individual contribution of $f_{\text {DBS}}$ and the second order Sobol’ index. The range of the second-order Sobol’ index gives the parameter interaction contributions to the output variance. (**F**) The second-order Sobol’ index of $A_{\text {DBS}}$ with its individual contribution in blue markers.

#### Optimised DBS parameters with respect to the network rhythmicity

We use the second objective function $\Phi _2$ based on the power spectrum of the mean membrane activity. The optimal DBS parameters will produce patterns according to the similarity of the DBS network rhythm compared to the healthy one. The optimal values for the position, frequency and amplitude were determined as $r=(x_0, y_0, z_0, A_{\text {DBS}}, f_{\text {DBS}}) = (-15.23, 3.37, 34.30, 253.74, 64.02)$. The optimal position and a network snapshot are depicted in Fig. 7A. The raster plot (Fig. 7B) shows sparse activity with periodicity around 60Hz due to the DBS effect. The mean network activity is depicted in Fig. 7C, jointly with healthy, pathological DBS in a position close to the refereed for OCD for comparison reasons. The spectrum (Fourier analysis) of the mean membrane activity $\overline{V}$ is shown in Fig. 7D. The power spectrum now shows the highest peaks at $\approx 55, 20$ Hz and secondary peaks at 125 and 192 Hz. Finally, two representative neurons are depicted in Fig. 7E. The neurons restore activity close to the healthy one.

#### Optimised DBS parameters with respect to the network rhythmicity and mean network activity

Next, we used a third objective function, $\Phi _3$, which optimises stimulation with respect to the network rhythmicity and the mean network activity. The optimal values for the position, frequency and amplitude were found to be $r=(x_0, y_0, z_0, A_{\text {DBS}}, f_{\text {DBS}}) = (-6.46, 4.87, -0.75, 367.89,109.19)$. The optimal stimulation position together with a network snapshot is depicted in Fig. 8A, The raster plot (Fig. 8B) shows a strong periodic activity at 110 Hz due to the high-amplitude, high-frequency DBS, similar to the previous optimisation case. The mean network activity is depicted in Fig. 8C, jointly with mean network activities under healthy and pathological states, as well as DBS optimal conditions and with DBS at the initial position. The resulting firing rate (using $\Phi _3$) shows a spike-like activity mainly related to the high-amplitude DBS stimulation, i.e. $A_{\text {DBS}} = 367.89$. The spectrum (Fourier analysis) of the mean membrane potential $\overline{V}$ is shown in Fig.8D. The power spectrum now shows the highest peak at $\approx 110$ Hz and a secondary peak at 220 Hz, i.e., as expected in the stimulation frequency range and its harmonics. Finally, the firing activity of two representative neurons is depicted in Fig. 8E. Interestingly, one neuron follows the DBS high frequency firing at 110 Hz, while the other is actually unaffected and firing at low frequency, showing that not all neurons are recruited into the stimulation.

### DBS induced synchronisation depending on the optimisation method

Our optimisation process introduces a strong synchronisation in the network activity, as illustrated in the raster plots of Figs. 6, 7, 8. We quantify the degree of network synchronisation using the *phase synchronisation index* $r_{\text {sync}}$^85,86^

42 $$\begin{aligned} r_{\text {sync}}(t)=\left| \frac{1}{N}\sum _{k=1}^{N}e^{i\theta _k(t)}\right| , \end{aligned}$$

where the phase $\theta _k(t)$ of the *k*-th neuron can be approximated linearly according to the following equation

43 $$\begin{aligned} \theta _k(t)=2\pi \frac{t-t_n}{t_{n+1}-t_n}+2\pi n, \end{aligned}$$

where, $t_n$ corresponds to *n*-th firing time of the *k*-th neuron and $t\in [t_n, t_{n+1}]$. The phase synchronisation index *r* describes the emerging macroscopic dynamics by taking the mean value of all phase populations (in exponential form, $e^{i\theta }$). The synchronisation index acts as an order parameter with range $r_{\text {sync}}\in [0,1]$, for example, in the case of perfect synchronisation, i.e. $\theta _1(t)=\theta _2(t)= \cdots =\theta _N(t)=\theta (t)$, the index can be written as

44 $$\begin{aligned} r_{\text {sync}}=\left| \frac{1}{N}\sum _{k=1}^{N}e^{i\theta _k}\right| =\frac{1}{N}N \left| e^{i\theta }\right| =1. \end{aligned}$$

Conversely, as $r_{\text {sync}} \rightarrow 0$, the phase dynamics become incoherent. We depict the synchronisation index for the three different optimisation methods in Fig. 9. The first optimisation method using $\Phi _1$ results in a strong synchronisation with maximum value $r_{\text {max}} = 0.67$. The second method, employing $\Phi _2$, results in a markedly reduced degree synchronisation with $r_{\text {max}} = 0.36$. Finally, for the third optimisation method, i.e., using $\Phi _3$, yields $r_{\text {max}} = 0.58$.

### Sensitivity analysis using Polynomial Chaos Expansion

We have performed a sensitivity analysis using polynomial chaos expansion (PCE) to study the sensitivity of the parameters involved in our model. For this analysis, we consider the parameters $I_0$, cortical excitation current and $g_{MM}$, i.e. conductance between MSN neurons, defined in the intervals [1.5, 5.0] and [0.0, 0.1], respectively. The parameter values are sampled following a uniform distribution. $n_s=(p+1)^d$ gives the number of samples, with $p=3$ the order of the polynomial chaos expansion and $d=2$ the number of parameters. We have 16 samples to run the model, each running in approximately 35 minutes. The macroscopic quantity of interest (QoI) is the firing rate of neurons.

Implementing the uncertainty quantification routine is based on the EasyVVUQ library^87^ in Python. The analysis approach is non-intrusive, i.e., the model is considered a black box. Fig. 10 presents the first-order and the higher-order Sobol’ indices obtained for the described configuration. As pointed out earlier in the methods section, Sobol’ indices correlate with the relative impact of any given parameter on the uncertainty in the output parameters. The simulation was performed for a time window from 200 ms to 400 ms. First, these indices were calculated, letting both parameters interfere with each other, and in a second step, the simulation was run for each parameter ($I_0$ and $g_{MM}$) independently. When both parameters can interfere with each other, it is visible that $g_{MM}$ has more impact than $I_0$ (Fig. 10A). When analysing the impact independently, $I_0$ remains below values of 0.4 (Fig. 10B), while $g_{MM}$ has a higher impact with an average value of 0.6 (Fig. 10C). Thus, although $I_0$ influences the firing rate, it is less significant than the variation of $g_{MM}$. While fluctuating over time, the first-order index $g_{MM}$ has an average value of 0.6, showing considerable importance in the model response (Fig. 10). One interpretation is that while cortical input, defined by $I_0$, generates the initial drive, $g_{MM}$ considerably boosts these cortical inputs. In other words, the internal conductance of MSN neurons plays a significant role in the network dynamics. It is conceivable that any pathological reduction of MSN activity or even dendritic remodelling in these cells will reduce the effect of glutamatergic and dopaminergic inputs onto the striatum and lead to abnormal striatal activity. Our results on sensitivity analysis also support this hypothesis.

We also performed the sensitivity analysis on the DBS state of the network for two DBS parameters, namely the amplitude of DBS $A_{\text {DBS}}$ and the frequency of DBS stimulation $f_{\text {DBS}}$. The (Fig. 10D) shows the first-order Sobol’ indices of these parameters for the firing rate. Figures 10E) and 10F) also show the second-order Sobol’ indices of $f_{\text {DBS}}$ and $A_{\text {DBS}}$. The stimulation amplitude, $A_{\text {DBS}}$ plays a major role in the firing rate compared to the stimulation frequency $f_{\text {DBS}}$.

## Discussion

We developed a biophysical network model for the striatum to explore the relationship between anatomical structure and neural activity, allowing us to calculate optimal DBS parameters based on spatio-temporal patterns produced by the model. The network construction was based on (a) an FDA-approved state-of-the-art human atlas^48^ (extracting coordinates for the striatal neurons), (b) on modified Hodgkin–Huxley equations for medium spiny neurons (MSN) and fast-spiking neurons (FSN)^46,47^, and (c) on complex network structures for neuronal connectivity^44,50,60–64^.

Depending on the model parameters, the network produced three spatiotemporal patterns, i.e. healthy, abnormal/pathological (presumably mirroring the situation in psychiatric disorders such as OCD and depression^23^), and DBS conditions. Simulating healthy conditions, the neuronal model produces macroscopic network activity with two main spectrum components, one peak on the $\theta$ rhythm (around 5 Hz) and the main peak at $\gamma$ frequency band (60 Hz, see Fig. 4). This $\gamma$ band activity is also observed in animals (rats) clinical studies of striatum^83,84^ during the movement initiation or for motivated behaviour and reward processing^83,84^

The model has shown reduced striatal activity by changing the conditions, specifically by reducing the cortico-striatal excitatory tone. This is also depicted in the spectrum diagram (see, Fig. 5), where, in contrast to healthy conditions, the highest peak appears at 4 Hz ($\theta$ band), while a secondary peak exists at 22 Hz ($\beta$ band) and a third one around 48 Hz. The reduced excitatory tone is explainable by a possible reduction of dopaminergic and glutamatergic inputs presumably occurring in neuropsychiatric conditions: Caravaggio et al.^23^ showed that chronic dopamine depletion ($>4$ months) produces decreases in striatal glutamate (consistent with the classical model of the basal ganglia). Dopamine reduction, in turn, has been observed in Major Depressive Disorder^17^. Furthermore, decreased functional connectivity (or decreased excitatory glutamatergic tone) between the sensorimotor cortex and dorsolateral striatum, and between dorsomedial striatum and dorsolateral prefrontal cortex has also been observed in PD patients^27,88^. Patients with obsessive-compulsive disorder, in turn, when carrying out a cognitive task, showed decreased responsiveness in the right medial and lateral orbitofrontal cortices, as well as in the right caudate nucleus (meaning to say, the cranial part of the striatum) when compared to controls^7^.

The proposed optimisation process was based on (a) the definition of objective functions (and thus possible biomarkers) that measure (or characterise) the network activity patterns and (b) the deviation of these biomarkers from the healthy state (the optimisation aiming to minimise this deviation). Our method estimates the optimal DBS parameters (including the following parameters: position, amplitude and frequency of the electrical signal) by a repetitive process, eventually aiming to restore or at least approximate healthy neuronal activity of the striatal network.

The first optimisation protocol relied only on the striatal network’s mean activity, $\langle a \rangle _T$, i.e., using the objective function $\Phi _1$ of Eq. (29). Following this optimisation procedure, the parameter $\langle a \rangle _T$ approximated neural activity parameters in the healthy state to a large degree. The optimal DBS parameters were found to be $r=(x_0, y_0, z_0, A_{\text {DBS}}, f_{\text {DBS}}) = (-9.62, 2.13, 11.22, 251.85, 99.54)$. This resulted in a very strong rhythmic activity with the synchronisation index to reach the value $r=0.67$. The network activity overcomes the sparse firing of the pathological state (with mean network activity to a normal state (see Fig. 6)), with the trade-off of very high rhythmicity. To alleviate this problem, we attempted a second optimisation approach guided mainly by power spectral analysis to specifically reduce the high rhythmicity, i.e., based only on Fourier analysis of neuronal action potentials, using $\Phi _2$. Indeed, this optimisation restored mean frequencies from $\theta$ in the pathological case to $\gamma$ very close to the healthy state. Additionally, the synchronisation levels are reduced to almost half, i.e. $r=0.36$, a 46% sync reduction. The downside of this approach is that the mean network activity Fig. 7B, C now falls much below normal activity (by  75%). A third optimisation attempt is then based on the combination of network rhythmicity and mean network activity. The optimal parameter in that case was found to be $r=(x_0, y_0, z_0, A_{\text {DBS}}, f_{\text {DBS}}) =(-6.46, 4.87, -0.75, 367.89,109.19)$. Importantly, this resulted in a 13% reduction in synchronisation (compared to $\Phi _1$), leaving this parameter closer to the healthy state but not quite meeting it, while again sufficiently raising firing from sparse to frequent and relatively close to a normal state (compare Fig. 8 C regarding network activity and D regarding the frequency power spectrum).

In conclusion, as *in vivo* studies on non-human primates^89^ suggest, there is a clear trade-off between frequency synchronisation and overall network activity when using DBS. Thus, given the apparent mutual inverse interdependence between an optimization strategy oriented on mean activation levels on the one hand and rhythmic synchronisation on the other, it appears that the effect of focusing on synchronisation level only (i.e. power spectrum using our approach $\Phi _2$) only leads to a moderate decrease in synchronicity (at lower frequency), at the cost of a nearly 75% loss of activation level. This disproportionate effect and the fact that using $\Phi _3$ as an optimisation method leads to close to normal activation levels (25-30% loss) and a power spectrum (and hence rhythmicity level) closest to healthy activity, and with reduced synchronisation levels compared to $\Phi _1$, lead us to assume that this might also be a functionally most effective strategy. The optimal strategy appears thus to be to raise mean activity in the first place while trying to minimise synchronicity as much as possible. Obviously, with such a strong external input as with DBS, some rhythmicity is always introduced, which is an artificial stimulus that does not appear in the normal context. One can, however, assume that *in vivo* in patients, this entrainment is dampened by the network and essentially low-pass filtered, and above all, inconsistently transferred within the entire circuitry^89^.

Different methods for finding optimal targeting positions for striatal DBS have been reported in the literature^90–93^. A clinical protocol is described in^93^, where critical parameters, e.g. the amount of current that is applied, the number of electrical pulses per second (frequency), the duration of these pulses (pulse width), and the amplitude of these pulses (similar to our parameters estimation), are tuned gradually depending on the positive performance of the patient. Other methods use hybrid approaches, combining clinical and computational methods, usually by correlating the activation of fibre bundles (calculating the volume of tissue activated) with patients’ optimal clinical response^90–92^. As proposed in^92^, axons (fibre) activation modulates neuronal network activity responsible for clinical improvement. However, fibre tracts and the volume of tissue activated do not provide any information on the reaction of the neuronal network (i.e. how the tissue activation or bundle activation modulates the neural activity). Only a recent publication^90^ studied the response of specific brain networks using an indirect biomarker, the intracranial electroencephalogram (EEG). In this paper, the authors identified hubs of critical white matter pathways (using tractography) connecting cortical and subcortical network regions relevant to the expression of depressive symptoms^90^. Our approximation constitutes a different, supplementary approach to the aforementioned studies based on cortical-striatal network activation. These two network parts are intricately entangled^94^; according to these authors, the relationship between neural activity in the cortex and striatum is “spatiotemporally precise, topographic, causal and invariant to behaviour, supporting, thus, a causal role of cortical inputs in driving the striatum”^94^. Finally, for completeness, we refer to multiple surgical targets for treating obsessive-compulsive disorder with deep brain stimulation (DBS)^95^: ant. cingulate cortex (-7.9,27.2,-7) and (-6.5,1.6,-4). Targets in Nucleus Accuben (-7.5, 10.8, -5) and ventral striatum / ventral capsule (-8.4, 3.5, -1), (-7.5, 15.3, -5).

Our study constitutes a computational approximation of the complex striatal network with assumptions and limitations. Regarding the assumptions, we used two types of neurons with simplified equations (see^46^). Furthermore, internal connectivity in the nuclei was assumed to take the form of small-world complex structures. This novel approach in basal ganglia modelling is reasonably justified in previous publications, both modelling and experimental^44,50,60–64^. As a limitation of the model, the exact structure of the connectivity on this microscopic level is unknown. Hence, how this can be modelled in the future remains to be clarified.

From a future modelling perspective, one important step forward will be the integration-connection of the striatal model with cortical areas, as well as with the other basal ganglia and thalamic nuclei. The new augmented model should contain synaptic plasticity effects (potentiation, depression), both known to be present at corticostriatal synapses, which strongly depend on the activation of dopamine receptors^1^. An integrative model will give new insight into possible mechanisms of DBS.

### Supplementary Information

Supplementary Information.

## Data Availability

The datasets generated and/or analysed during the current study are available in the gitHub SFB-Elaine repository: https://github.com/SFB-ELAINE
