# Supplementary material for: Utilising activity patterns of a complex biophysical network model to optimise intra-striatal deep brain stimulation
Source: Sci Rep. 2024 Aug 14;14:18919. doi: 10.1038/s41598-024-69456-7 (PMC11324959; doi:10.1038/s41598-024-69456-7)
Supplement: Supplementary file 1 — Supplementary Information. [file 41598_2024_69456_MOESM1_ESM.pdf]

Utilising activity patterns of a complex biophysical network model to optimise intra-striatal deep brain stimulation

Konstantinos Spiliotis, Revathi Appali, Anna Karina Fontes Gomes,
Jan Philipp Payonk, Simon Adrian, Ursula van Rienen,
Jens Starke, Rüdiger Köhling

1 Supplementary material

1.1 Transition from healthy to pathological states with respect to cortico-striatal current intensity

For each value of the parameter I_0, we calculate the area under the spectrum curve in the range of [0, 80] Hz, resulting in the value A_{tot}. Furthermore, we compute the area A_k, $k = 1, 2, 3, 4$, for each rhythm. These include the 30-80 Hz γ band, 12-30 Hz β band, 8-12 Hz α band, and the 0.5-8 Hz δ and θ bands. The relative power is defined as the ratio: $\frac{A_k}{A_{\mathrm{tot}}}$. Starting from higher values of cortico-striatal current, specifically $I_0 = 5$ μA/cm^2, and decreasing the intensity of the current until $I_0 = 3$ μA/cm^2, we observed that the behaviour of the γ band activity remained constant. This is evidenced by the fact that the area under the curve remained at a constant value of approximately 0.8 of the total rhythm. The γ band rhythm undergoes a significant reduction between $I_0 = 3$ μA/cm^2 and $I_0 = 2.4$ μA/cm^2. At lower values of I_0, namely $I_0 < 2.4$ μA/cm^2, the β, δ, and θ rhythms emerge as the dominant frequencies bands.

1.2 Improved optimisation of DBS with respect to the activity of a specific targeted area (dorsal striatum)

In our network modularity analysis, a partitioning of the striatum into six major areas emerges (see Fig. 3 of which the dorsal striatum is the most strongly affected by stimulation. For this reason, in the next step, we again optimised with respect to the mean activity, i.e. using Φ_1. This time, however, we only

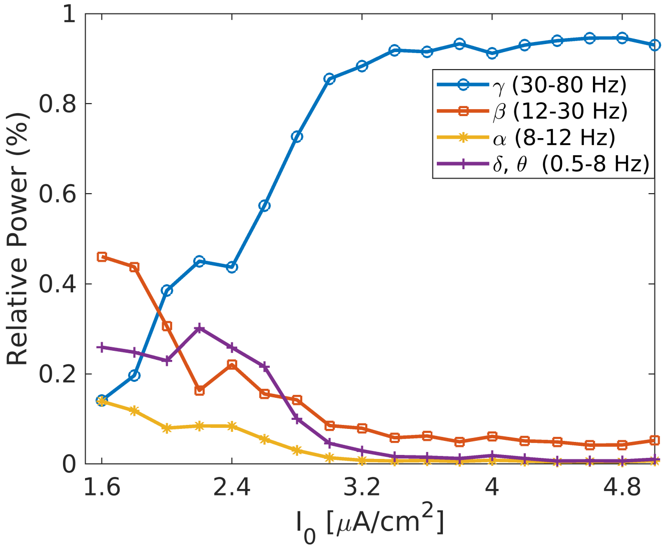

Figure 1: **Rhythmicity of network with respect to the parameter** I_0. For each value of the parameter I_0, we calculate the area under the spectrum curve in the range of [0 80]Hz, given as A_{tot}. We compute also the area A_k for each rhythm i.e., for 30-80 Hz γ, 12-30 Hz β, 8-12 Hz α and finally 0.5-8 Hz δ, θ rhythm. The relative power is defined as the ratio: $\frac{A_k}{A_{tot}}$.

restricted the analysis to area 2 in Fig. 3. Under these conditions, an outcome similar to the optimisation process based on the combination of network mean activity, and network rhythmicity emerges. Thus, under these conditions, the optimal values for the position, frequency and amplitude were found to be $r = (x_0, y_0, z_0, A_{\rm DBS}, f) = (-11.43, 4.26, 20.81, 200.16, 98.94)$. The optimal position together with a network snapshot is depicted in Fig. 2A, The raster plot (Fig. 2B) shows activity around 100 Hz due to the DBS effect. The mean network activity is depicted in Fig. 2C, again with the results from healthy and pathological (abnormal) conditions. The optimised condition (with respect to the phase, blue thick line) approximates the healthy state (red line). The spectrum (Fourier analysis) of the neuronal firing rates r_T is shown in Fig.2D, again showing 100 Hz and harmonics as peaks, as expected. Finally, two representative neurons are depicted in Fig.2E. The neurons show restored activity resembling the healthy state.

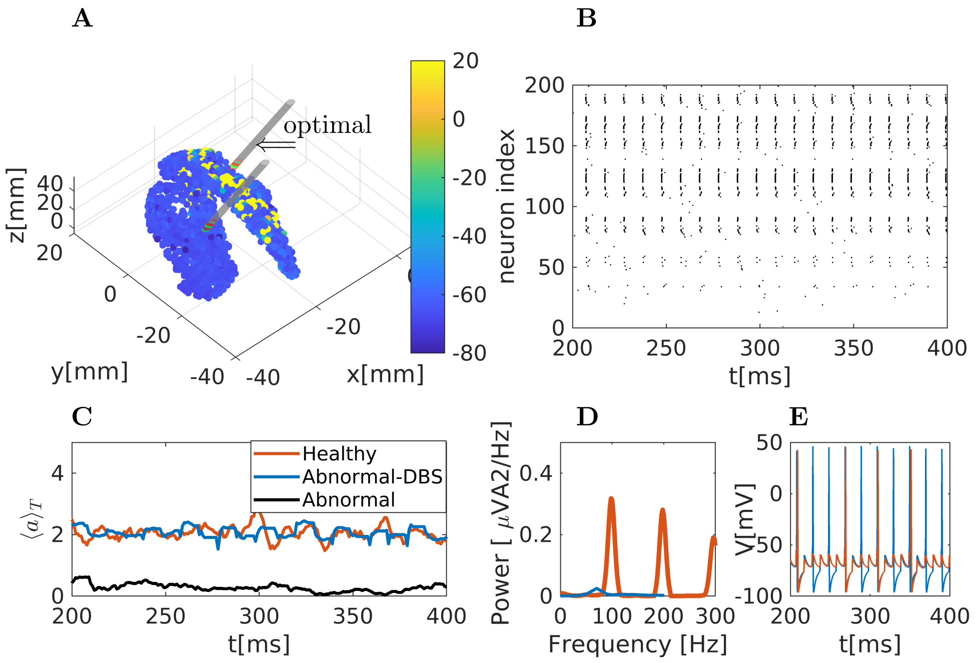

Figure 2: **Optimised DBS activity for a specific targeted striatum area** **A** Snapshot of the striatum activity during DBS. Colour coding is according to the membrane potential (in mV). Two electrodes are included. One corresponds to the optimal position, marked with an arrow, while the second displays the initial position. **B** Raster plot representation. Black dots represent activated neurons, and the activity is synchronised. **C** Mean activity of the striatal network. Blue thick line (mean activity) resulting under optimal DBS positioning conditions. We also compare mean activities under healthy, pathological, and DBS conditions with stimulation in the initial, non-optimised position. **D** Power spectrum of the firing rate. **E** Two representative neurons were simulated using a stimulation set at optimal DBS conditions.
